# Physics-based modeling and friction parameter identification of a proportional spool valve

**DOI:** 10.1038/s41598-026-46361-9

**Published:** 2026-03-31

**Authors:** Marian Ledvoň, Lumír Hružík, Adam Bureček, Tomáš Polášek, David Kolář

**Affiliations:** Department of Hydromechanics and Hydraulic Equipment, VSB-Technical University of Ostrava, Ostrava, Czech Republic

**Keywords:** Proportional directional valve, Step response, Frequency response, Physics-based model, Friction force, Parameter identification, Engineering, Mathematics and computing

## Abstract

This paper presents the development of a physics-based model of a proportional directional valve with the aim of describing its dynamic behavior while accounting for the influence of friction forces acting on the spool. The model is derived from the spool’s equation of motion, and in order to achieve accurate parameter tuning, an experimental analysis of the individual forces acting on the spool was carried out. Particular attention is given to the parameters of the friction force, as this component represents a key factor affecting the dynamic response of the system. Due to the difficulty of direct measurement, the identification of friction parameters was performed through a combined numerical–experimental comparison of the system’s response to step control inputs. A significant advantage of the proposed model lies in its capability to parameterize the individual force components independently, which facilitates straightforward adaptation to different operating conditions or valve types. For experimental validation, a series of dynamic measurements was conducted on a specialized hydraulic test rig. The acquired data were used both to identify the friction model parameters and to validate the simulation model. A comparison of the simulation results with experimental data demonstrates good agreement across a wide range of operating conditions. The proposed model therefore provides a flexible and accurate tool for predicting the behavior of proportional directional valves, applicable both in the design of control algorithms and in the optimization of valve construction.

## Introduction

Proportional directional valves are widely employed in hydraulic control systems due to their ability not only to control the flow direction but also to provide smooth flow regulation^1–8^. Their dynamic characteristics directly affect the stability, accuracy, and efficiency of the overall hydraulic system^9^. An accurate description of valve behavior is therefore essential both for the design of control algorithms and for optimizing valve construction. The dynamic response of spool-type valves arises from the interaction of several physical phenomena, including hydrodynamic forces^10,11^, the electromagnetic force of the actuator^12,13^, and the mechanical effects of springs and friction. A comprehensive overview of design variations and research approaches is provided in the review by Tamburrano et al. (2018)^14^, which summarizes the industrial state of the art in direct-operated proportional spool valves and identifies key areas for optimization, including fluid characteristics and spool position control systems. Among these factors, friction between the spool and the valve body plays a key role in shaping the dynamic response, particularly in systems operating under variable conditions.

Numerical modeling of proportional spool valves has been the subject of extensive research, with approaches ranging from purely empirical models to detailed simulations based on computational fluid dynamics (CFD)^15–22^. Empirical models are computationally efficient but often lack the ability to reliably predict valve behavior outside the tested operating range. Conversely, CFD simulations provide highly detailed insight into flow fields and interactions within the valve components, but at the expense of significant computational effort and complex input data preparation, as confirmed by Li et al.^23^.

Physics-based models with a parametric formulation represent a suitable compromise, as they capture essential dynamic phenomena while maintaining reasonable computational demands. Such models provide deeper insight into the mechanisms influencing valve behavior and allow independent parameterization of the contributing force components^24^. The foundations of mathematical modeling of electrohydraulic and proportional spool valves have been well established in classical works by Merritt^25^ and Manring^26^, who formulated dynamic equations describing spool motion, actuator dynamics, and flow-induced forces based on physical principles. Building on these foundations, numerous studies have addressed the modeling of proportional directional valves using physics-based approaches, with particular attention to nonlinear effects such as friction, electromagnetic actuation, and flow-dependent forces^27,28^. The benefits of these approaches are demonstrated by Lisowski et al.^29^, who addressed pressure-compensation effects through design modifications and analysis of hydrodynamic forces in proportional valves. Similarly, Wang et al.^30^ applied spool-geometry optimization to reduce steady-flow torques, thereby supporting design strategies aimed at improving valve dynamic response. Zhang et al.^31^ further highlighted the importance of analyzing both static and dynamic characteristics, including dead-zone and hysteresis, in servo valves for robotic applications, underscoring the need for accurate modeling of nonlinear effects. As a result, these models can be easily adapted to specific operating conditions or different valve configurations. A critical challenge, however, remains the accurate parameterization of forces that are difficult to measure directly.

Particular attention must be given to the friction force between the spool and the valve body, which strongly influences the system’s dynamic response^32^. Friction modeling is complicated by its nonlinear nature, dependence on operating conditions, and potential interactions with other dynamic phenomena. Although several friction models (such as Coulomb, Stribeck, and LuGre) have been successfully applied in hydraulic and mechatronic systems^33–35^, their parameters must be carefully identified to ensure that simulations match real behavior. Direct measurement of friction in an operating valve is difficult, which often leads to the use of indirect identification methods based on comparing numerical simulations with experimentally recorded system responses^36,37^.

The objective of this study is to develop a physics-based model of a proportional spool valve that explicitly incorporates the individual force components acting on the spool and enables their independent parameterization. Special attention is given to friction model parameter identification, as friction represents the dominant factor influencing valve dynamics. Another objective is to perform experimental measurements of the valve’s dynamic characteristics and use these data for model parameters identification, as spring stiffness and mass of the moving elements. The proposed model is subsequently validated by comparison with experimental results over a wide range of operating conditions. The simulation model demonstrates good agreement with measurements while offering flexibility for adaptation to different valve types and operating environments.

## Equation of motion of the proportional directional valve

### Tested valve description

For the purposes of this study, a proportional directional valve designated PRL2-06-32-0-24 manufactured by Argo Hytos was selected. The valve is a direct-operated 4/3 proportional spool valve. Such valves are primarily applied in linear and rotary hydraulic drives, where continuous control of the piston rod motion or shaft rotation is required.

**Fig. 1:**
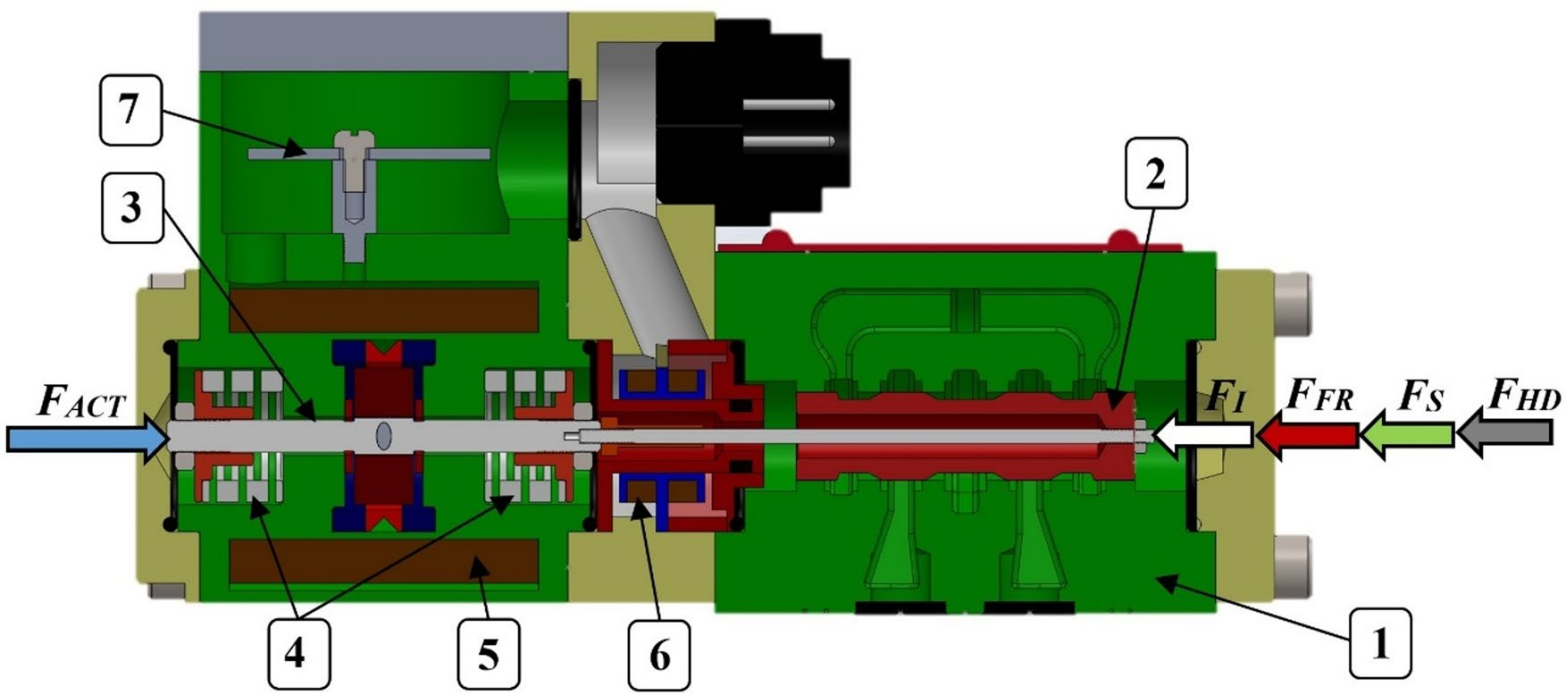
Cross-section of the 3D model of the proportional directional valve showing individual components and forces acting on the spool: (1) valve body, (2) spool, (3) linear motor armature, (4) springs, (5) linear motor coil, (6) spool position inductive sensor, (7) integrated electronics^39^.

From a component perspective, the valve can be divided into three main sections. The hydraulic section consists of a cast-iron housing (1) with a precisely fitted spool (2). The spool is designed with sharp-edged geometry and features zero overlap in the central position. The actuation section is formed by a linear motor. Centering of the linear motor armature (3), which is rigidly connected to the spool via a threaded joint, is ensured by two springs of equal stiffness (4). Depending on the magnitude and direction of the electric current supplied to the linear motor coil (5), the armature—and consequently the spool—is displaced from its neutral position. In the event of a power loss, the armature together with the spool is returned to the central position by the springs. Between the armature and the spool, an inductive position sensor (6) is integrated. This sensor, which is built directly into the valve body, provides real-time information on the actual spool position and thus forms the third main section of the valve. The valve also includes integrated electronics (7) that process the spool position feedback and allow adjustment of the neutral spool position. The measured spool position is further transmitted to the electronic control unit designed specifically for PRL2 proportional directional valves. A sectional 3D model of the proportional directional valve with the identified components is presented in Fig. 1, while the technical specifications of the tested valve are summarized in Table 1.

**Table 1:** Technical specifications of the PRL2 proportional directional valve.

Parameter	Unit	Value
Nominal size	–	06
Max. operating pressure	MPa	35
Nominal flow ∆*p* = 7 MPa	dm^3^ min^− 1^	32
Nominal flow ∆*p* = 1 MPa	dm^3^ min^− 1^	12.5
Hysteresis	%	< 1
Sensitivity	%	< 0.5
Internal leakage	dm^3^ min^− 1^	< 1.5
Viscosity range	mm^2^ s^− 1^	20–400

In its most basic representation, the dynamic behavior of proportional directional valves can be modeled as a proportional element exhibiting second-order inertia. This formulation captures the essential inertial and damping characteristics of the system and is commonly employed for control design, regulator tuning, and estimation of the system’s temporal response. The corresponding differential equation can be expressed as (1):

1 $$\:{T}^{2}\ddot{x}+2\xi\:T\dot{x}+x=kU,$$

where *T* is the time constant, $\:\xi\:$ is the relative damping ratio, *x* is the spool stroke, *k* is the valve gain and *U* is the control voltage^25,26^.

While the aforementioned representation is computationally efficient and well-suited for basic simulation tasks, it lacks the capability to provide a physical interpretation of the individual forces acting on the spool. Consequently, it is not suitable for design optimization or for predicting system performance under variations in structural parameters (e.g., spool mass, spring preload, friction). To achieve a more accurate description of the spool dynamics, a physics-based model derived from Newton’s second law is required^25,26^. In this framework, the spool is treated as a rigid body with lumped mass. When actuated by the electromechanical transducer (represented in this case by a linear motor) the spool is subjected to axial forces, as illustrated in Fig. 1. The dynamic behavior of the valve can be expressed by the following equation of motion (2):

2 $$\:{F}_{I}\left(\ddot{x}\right)+{F}_{FR}\left(x,\dot{x}\right)+{F}_{S}\left(x\right)+{F}_{HD}\left(x,\dot{x}\right)={F}_{ACT}(x,I),$$

where *F*_*I*_ is the inertial force of the spool, *F*_*FR*_ is the friction force, *F*_*S*_ is the spring force, *F*_*HD*_ is the hydrodynamic force, *F*_*ACT*_ is the force generated by the linear motor required for spool stroke, and *I* is the control current supplied to the linear motor coil^38^.

The aforementioned model accounts for the electromechanical force generated by the linear motor coil, which depends on both the spool position and the control current. Additionally, it incorporates the spring force, the inertial force associated with the mass of the moving elements, the hydrodynamic force induced by the fluid flow, and the friction force.

This physics-based model provides the foundation for numerically simulating the spool dynamics of the proportional directional valve. A key advantage of this approach is that, once all known force contributions are incorporated into the model, the resulting equation contains only one unknown term, the friction force *F*_*FR*_, whose direct experimental measurement is technically challenging. Consequently, the proposed model serves as the basis for an inverse problem, wherein the friction force magnitude is inferred from the measured dynamic response of the system. This approach enables the back-calculation of friction model parameters, which is presented in the section dedicated to friction force identification.

In the subsequent sections, the individual force components of the motion equation are examined in detail.

### Linear motor force

The electromechanical actuator of the investigated proportional directional valve is a linear motor that produces the spool’s translational motion in response to the control input current. Accurate mathematical modeling and simulation of the valve’s dynamic behavior require knowledge of the relationship between the input current applied to the linear motor coil, the armature stroke, and the resulting output force acting on the spool. To establish this relationship, an experimental analysis of the linear motor’s force characteristic was conducted with the aim of determining the dependence of the output force *F*_*ACT*_ on the input current *I* and spool stroke *x*.

The force characteristic was measured under static conditions using a force sensor with a measurement range of ± 50 N and a linearity error of 0.5%. The linear motor was supplied with defined current values over the range *I* = (− 2–2) A, while the armature stroke was mechanically constrained. Measurements were performed sequentially at discrete armature positions spanning the entire working stroke of the spool, i.e., *x* = (− 0.57–0.57) mm.

The resulting force characteristic was recorded as a matrix, where each entry corresponds to the output force *F*_*ACT*_ measured by the force transducer for a given pair of input current *I* and armature stroke *x*. For subsequent analysis, only the data corresponding to one direction of armature stroke were utilized, as experimental verification confirmed that the characteristic is symmetric with respect to the stroke direction. This symmetry was validated by comparing measurements for positive and negative stroke values under identical current inputs.

**Fig. 2:**
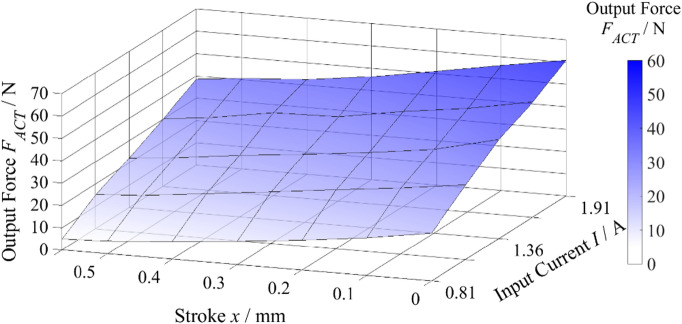
Force characteristic of the linear motor.

The measured data are depicted in Fig. 2, showing the dependence of the linear motor output force on both the input current and the armature stroke. The results demonstrate that, at a constant armature stroke, the output force exhibits an increasing trend with higher control current. Conversely, for a constant input current, the generated force decreases as the armature stroke increases.

The experimental characterization of the linear motor’s force response constitutes a critical input for parameterizing the actuator force term within the valve model. The acquired dataset describes the relationship between output force, input current, and armature stroke, thereby facilitating an accurate representation of the actuator’s behavior in the computational framework. The characteristic is stored in matrix form and implemented in the numerical simulation as an input to the spool motion equation, with the force value interpolated based on instantaneous spool stroke and input current. This method enables the model to incorporate the actual actuator force, which in turn supports a reliable identification of the remaining unknown parameters (most notably the friction forces) while enhancing the overall accuracy of the valve’s dynamic behavior simulation. The measured static force characteristic of the linear motor includes its internal friction; however, this friction is not explicitly modeled in the dynamic simulations, as the dominant contribution to system damping originates from the interaction between the spool and the valve body.

### Spring force

**Fig. 3:**
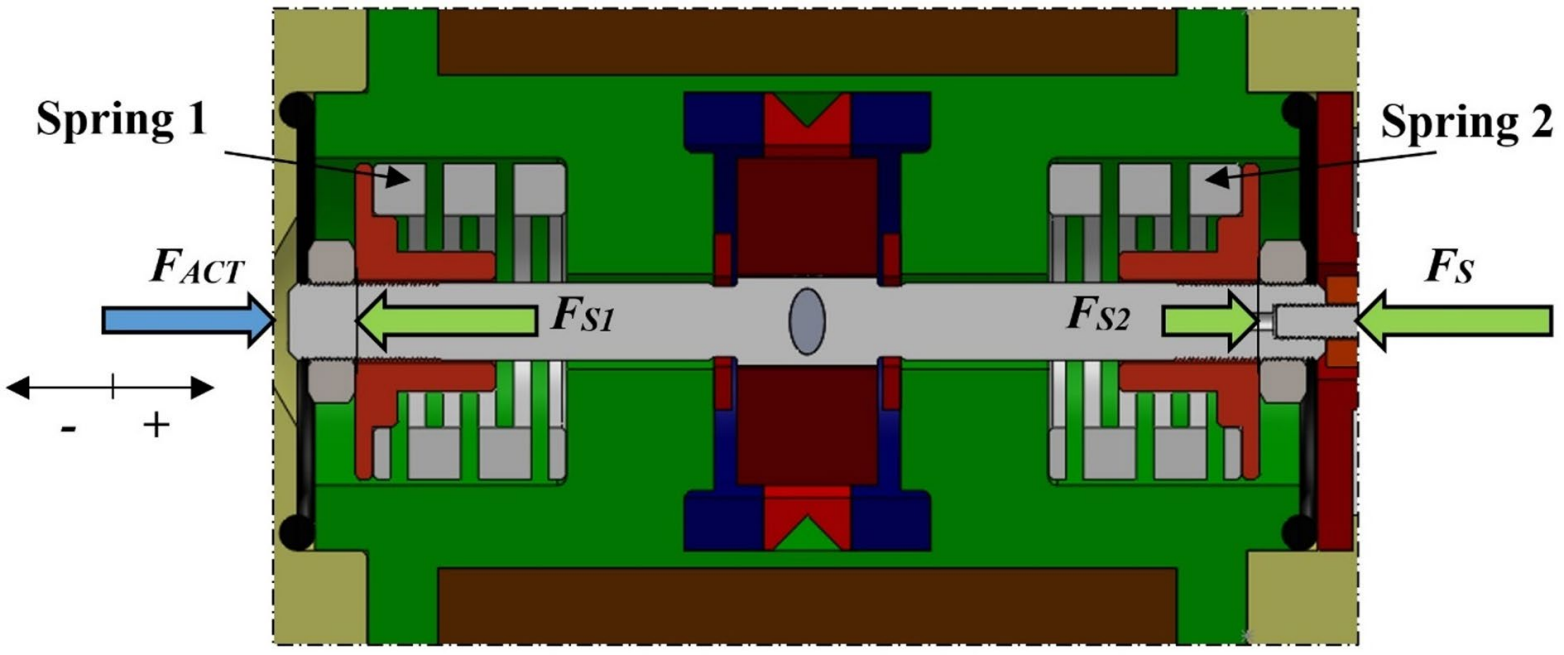
Spring arrangement and decomposition of forces generated by preloaded springs.

In the mechanical layout of the investigated proportional directional valve, two identical compression springs are mounted symmetrically and act in opposition, each attached to the respective ends of the linear motor armature. The armature is mechanically linked to the spool via a threaded joint. The springs generate a restoring force that depends on the instantaneous spool stroke *x* relative to its neutral position. This arrangement is schematically illustrated in Fig. 3, which shows the individual spring force components *F*_*S*1_ and *F*_*S*2_​ as well as the resultant spring force *F*_*S*_​, included in the system’s equation of motion. The notation *F*_*ACT*_ denotes the direction of the force exerted by the armature on the springs.

**Fig. 4:**
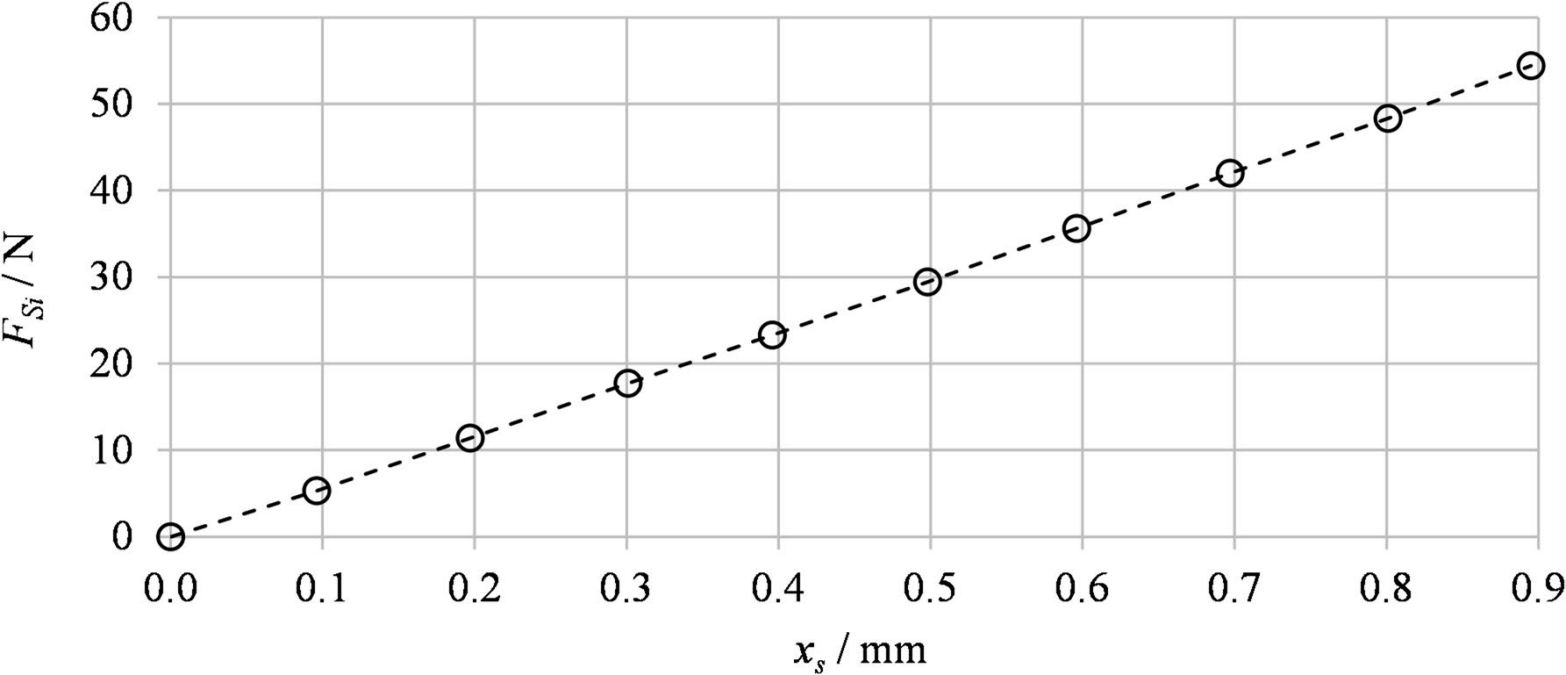
Experimentally measured dependence of the single-spring force *F*_*Si*_ on compression *x*_*S*_.

For a precise description of the spring force contribution, an experimental procedure was carried out to determine the spring stiffness *k*_*S*_. During the experiment, the force applied to the spring was recorded simultaneously with its compression over the range *x*_*S*_ = (0–0.9) mm. Linear regression of the measurement data yielded a stiffness value of *k*_*S*_​ = 60.9 N mm^− 1^, confirming a linear characteristic across the entire stroke range. The spring preload was also measured as *x*_*S*0_ = 0.7 mm, corresponding to the predefined compression at the neutral spool position (*x* = 0 mm). The experimentally obtained dependence of the single-spring force *F*_*Si*_ on its compression *x*_*S*_ is presented in Fig. 4. The results confirm a linear force characteristic within the investigated compression range, thereby supporting the identification of the spring stiffness *k*_*S*_.

The compression force of an individual spring *F*_*Si*_ is described by (3):

3 $$\:{F}_{Si}={k}_{S}\left({x}_{S0}\pm\:x\right),$$

where *k*_*S*_ denotes the spring stiffness, *x*_*S*0_ is the preload and *x* is the instantaneous spool stroke^25,26^.

Considering the measured characteristics and the known mechanical configuration, the spring force was incorporated into the model as the difference between the forces of the two opposing springs (4):

4 $$\:{F}_{S}={F}_{S2}-{F}_{S1}.$$

Although the spring force formulation is based on well-established linear elasticity principles, the following analytical expressions were derived in this work to account for the specific preload conditions and the stroke-dependent behavior of the proportional valve spool. Consequently, the total spring force *F*_*S*_​ is formulated as a function of spool stroke *x*, spring stiffness *k*_*S*_​ and preload *x*_*S*0_​. The model distinguishes between positive, neutral, and negative spool strokes based on whether the preload is exceeded. The analytical expressions (5)–(7) employed to represent the spring-generated force are presented in the following section.

5 $$\:x > 0\quad \:{F_S} = {F_{S1}} - {F_{S2}}\left\{ {\begin{array}{*{20}{c}}{\:\:{k_S}\left( {{x_{S0}} + x} \right) - {k_S}\left( {{x_{S0}} - x} \right)\quad \:x < {x_{S0}}\:} \\ {\:{k_S}\left( {{x_{S0}} + x} \right)\quad \:x \geqslant \:{x_{S0}}} \end{array}} \right.$$

6 $$\:x = 0\:\quad {F_S} = {F_{S2}} - {F_{S1}} = 0\quad \:{F_{S2}} = {F_{S1}}$$

7 $$\:x < 0\quad \:{F_S} = {F_{S2}} - {F_{S1}}\left\{ {\begin{array}{*{20}{c}}{\:\:{k_S}\left( {{x_{{\mathrm{S}}0}} + \left| x \right|} \right) - {k_S}\left( {{x_{{\mathrm{S}}0}} - \left| x \right|} \right)\quad \:\left| x \right| < {x_{S0}}\:} \\ {\:{k_S}\left( {{x_{S0}} + \left| x \right|} \right)\quad \:\left| x \right| \geqslant \:{x_{S0}}} \end{array}} \right.$$

**Fig. 5:**
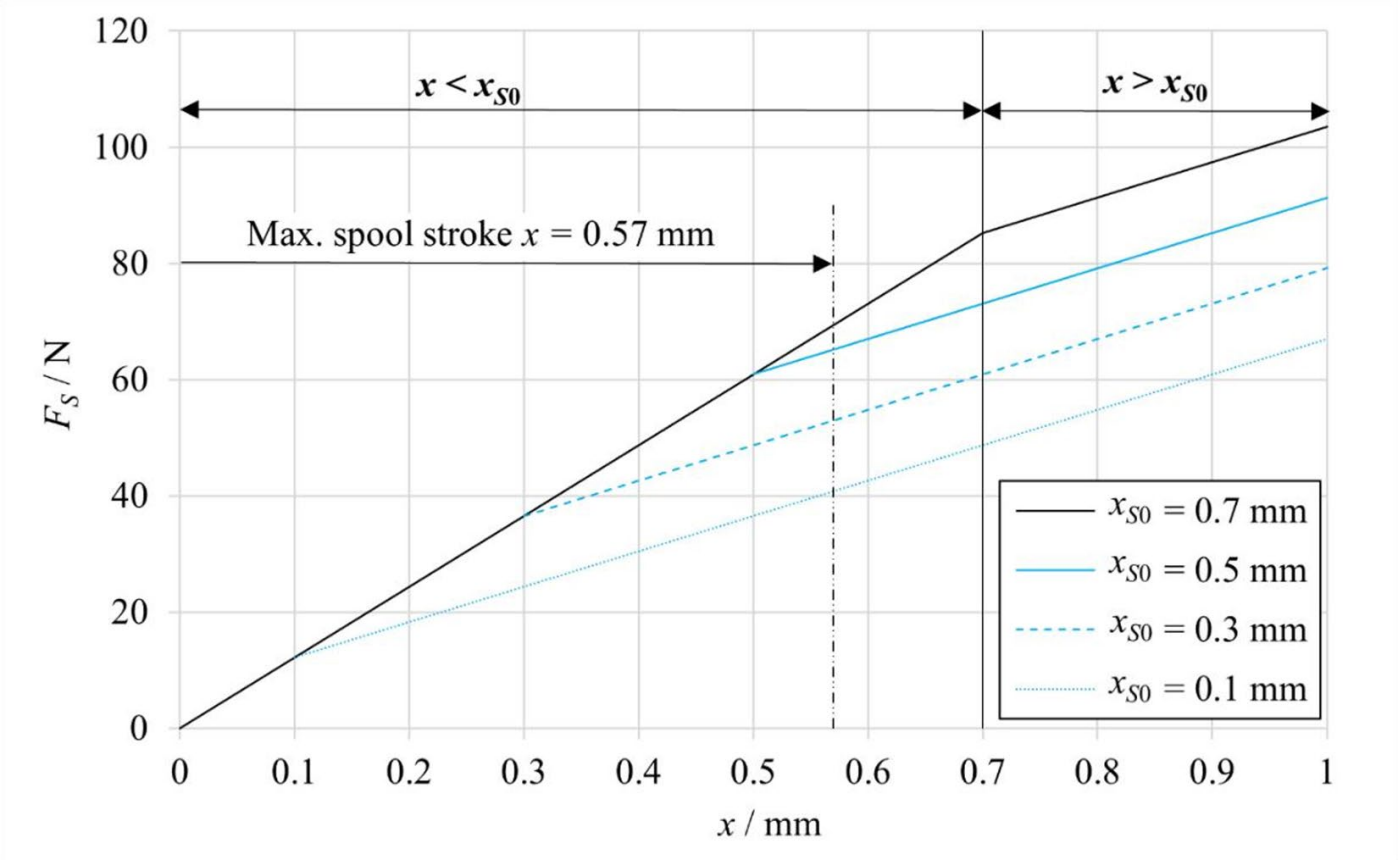
Dependence of the resulting spring force on spool stroke for different spring preload values *x*_*S*0_.

The derived expressions accurately reflect the influence of spring preload on the resulting force profile, particularly near the stroke limits, where one of the springs may become fully unloaded (i.e., reach its free length and no longer generate any force). As shown in Fig. 5, for the analyzed valve configuration with a spring preload of *x*_*S*0_ = 0.7 mm, and a maximum operational spool stroke of *x*_*MAX*_ = 0.57 mm, neither spring becomes fully unloaded within the operating range. Consequently, the spring-generated force remains linear across the entire stroke. The figure also compares the resulting force *F*_*S*_​ for various values of the preload *x*_*S*0_​. The comparison reveals that, when the preload is smaller than the maximum spool stroke, the force characteristic exhibits a pronounced piecewise behavior. This effect results from the unilateral disengagement of one spring, which ceases to exert force once its preloaded length has been exceeded. In the mathematical model, this phenomenon is incorporated through Eqs. (5)–(7), which treat the spring preload as an adjustable parameter. This formulation enables analysis of how mechanical spring settings affect system stability and dynamic response under different control scenarios.

Based on the conducted experimental measurements, the spring stiffness and initial preload were established, and their effects were incorporated into the simulation model as a piecewise linear force characteristic dependent on the spool stroke. The configuration of oppositely preloaded springs produces a resultant force that substantially influences the steady-state spool position, especially in open-loop conditions. The model permits variable adjustment of the preload values, allowing simulation of different actuator operational configurations. This feature thus represents a critical interface between the mechanical assembly and the dynamics of spool position control.

### Inertial force

To accurately represent the spool dynamics within the simulation model, the effective mass of the moving assembly was determined and incorporated into the computation of the inertial force. The total moving mass comprises the spool, the connecting rod, the linear motor armature, and one-third of the combined mass of both springs. The inclusion of one-third of the spring mass follows the conventional treatment of mechanical oscillatory systems, where only a portion of the spring mass is attributed to the moving element^26^. The individual component masses were measured using a calibrated laboratory-grade digital scale to ensure high accuracy and repeatability. The resulting total moving mass was found to be *m* = 45.1 g. Within the simulation framework, this parameter is defined explicitly to facilitate straightforward modifications and parametric studies. Consequently, any design changes to the spool (such as geometry optimization or material substitution) can be readily reflected in the model to capture the corresponding dynamic behavior. The mass is treated as a lumped parameter and enters the equation of motion through the inertial force term, expressed as (8):

8 $$\:{F}_{I}=m\cdot\:a,$$

where *a* denotes the spool acceleration and *m* is the total mass of the moving assembly^25,26^.

### Friction force

Friction is an inherent phenomenon in all mechanical systems where relative motion occurs between components. While it can be beneficial in certain applications (e.g., braking systems), in systems requiring precise position and velocity control (such as proportional valves) it constitutes a major source of nonlinearities and uncertainties. Friction in these contexts often degrades positioning accuracy, induces hysteresis, causes response delays, and adversely affects the overall dynamic performance of the system. The frictional force acting within mechanical assemblies is inherently complex, depending not only on the relative velocity but also on the direction of motion, applied load, temperature, and other influencing factors. In engineering practice, several mathematical models of varying complexity are employed to describe its behavior.

#### Basic friction model

Coulomb friction, which is independent of velocity and acts as a constant opposing force determined by the direction of motion, can be expressed as (9):

9 $$\:{F}_{C}={{f}_{FR}F}_{N}sign\left(v\right),$$

where *F*_*N*_​ denotes the normal force, *f*_*FR*_ is the coefficient of friction and *v* represents the relative velocity^35^.

Viscous friction, which is directly proportional to velocity, is described by (10):

10 $$\:{F}_{V}={f}_{V}v,$$

where *f*_*V*_​ denotes the viscous damping coefficient^35^.

The Stribeck effect, characterizing the reduction in frictional force at low velocities, can be formulated as a velocity-dependent function and is herein retained in its general form *F*_*ST*_(*v*)^35^. The overall friction force, incorporating all three contributions, is then expressed as (11):

11 $$\:{F}_{FR}\left(v\right)={{f}_{FR}F}_{N}sign\left(v\right)+{f}_{V}v+{F}_{ST}\left(v\right).$$

#### Advanced friction model: LuGre model

To achieve a more accurate representation of frictional effects, the so-called bristle model (LuGre) is employed, in which the contact surfaces are microscopically modeled as elastic bristles. As relative motion occurs, these bristles deflect and undergo stochastic slip due to random asperity interactions and surface irregularities. The model dynamics are described by the following set of equations.

The evolution of the average bristle deflection is governed by (12):

12 $$\:\frac{dz}{dt}=v-\frac{\left|v\right|}{g\left(v\right)}z,$$

where *z* denotes the bristle deflection, *v* is the relative velocity and *g*(*v*) is a function describing the Stribeck effect. The function *g(v)* je is strictly positive and depends on various factors such as material properties and temperature. As the relative velocity increases *g*(*v*) monotonically decreases from its static value *g*(0), capturing the Stribeck phenomenon^33,34^.

The friction force generated by the bending of the bristles is modeled as (13):

13 $$\:{F}_{FR}={k}_{B}z+c\frac{dz}{dt}{+f}_{V}v,$$

where *k*_*B*_ represents the bristle stiffness, *c *is the damping coefficient, *f*_*V*_ is the viscous friction coefficient and *v* is the relative velocity^33,34^.

The above model, defined by Eqs. (12) and (13), relies on the function *g(v)* and the parameters *k*_*B*_, *c* and *f*_*V*_. The steady-state relationship $\:{k}_{B}g\left(v\right){+f}_{V}v$ can be identified experimentally from constant-velocity friction measurements. The Stribeck effect itself is typically parameterized as (14):

14 $$\:{k}_{B}g\left(v\right)={F}_{C}+\left({F}_{ST}-{F}_{C}\right){e}^{-{\left(\frac{v}{{v}_{ST}}\right)}^{2}},$$

where *F*_*C*_ denotes the Coulomb friction force, *F*_*ST*_ is the breakaway (static) friction force, and *v*_*ST*_ is the Stribeck velocity^33,34^.

Under steady velocity conditions, the friction–velocity characteristic is modeled by (15)^33,34^.

15 $$\:{F}_{FR}\left(v\right)={k}_{B}g\left(v\right)sign\left(v\right)+{f}_{V}={F}_{C}sign\left(v\right)+\left({F}_{ST}-{F}_{C}\right){e}^{-{\left(\frac{v}{{v}_{ST}}\right)}^{2}}sign\left(v\right)+{f}_{V}v.$$

For numerical implementation, a modified version of the LuGre model is adopted to ensure a smooth transition between static and dynamic friction and to accurately capture the typical nonlinear frictional behavior observed at low velocities. This variant is formulated as (16), (17) and (18):

16 $$\:{F}_{FR}=\sqrt{2e}\left({F}_{ST}-{F}_{C}\right){e}^{-{\left(\frac{v}{{v}_{{ST}}}\right)}^{2}}\frac{v}{{v}_{ST}}+{F}_{C}\cdot\:{tanh}\left(\frac{v}{{v}_{Coul}}\right)+{f}_{V}v$$

17 $$\:{v}_{ST}={v}_{BR}\sqrt{2}$$

18 $$\:{v}_{Coul}={v}_{BR}/10,$$

where *F*_*FR*_ is the friction force, *F*_*C*_ is the Coulomb friction force, *F*_*ST*_ is the breakaway friction force, *f*_*V*_ is the viscous friction coefficient, *v* is the spool’s relative velocity, *v*_*ST*_ is the Stribeck velocity, *v*_*Coul*_ is the characteristic Coulomb velocity, *v*_*BR*_ is the breakaway friction velocity, and *e* denotes Euler’s number^42^.

### Hydrodynamic force

When the spool is displaced from its neutral (center) position, fluid flow through the valve generates a hydrodynamic force *F*_*HD*_ acting on the spool. This force consists of two components: a steady-state component *F*_*HDS*_ and a transient component *F*_*HDT*_​.

The steady-state hydrodynamic force *F*_*HDS*_ arises from the change in axial momentum of the fluid as it flows across the spool. Regardless of the direction of fluid flow, this component always acts in the closing direction of the valve orifice. It can be expressed as (19):

19 $$\:F_{HDS}=\rho \frac{Q^2}{S_p}\cos(\theta)$$

where *ρ* is the fluid density, *S*_*p*_ is the throttling cross-sectional area, *Q* is the volumetric flow rate, and $\:\theta$ is the angle of the fluid jet with respect to the spool axis^26,40^.

The transient hydrodynamic force *F*_*HDT*_​​ is generated by changes in the velocity of the fluid surrounding the spool within the valve chamber. Depending on the sign of $\frac{\partial Q}{\partial t},$ this component may act either in the closing or the opening direction of the orifice. Based on Newton’s second law, its magnitude is given by (20):

20 $$F_{HDT}=\rho L \frac{\partial Q}{\partial t}$$

where *L* is the axial distance between the inlet and outlet fluid streams^26,40^.

The resulting hydrodynamic force acting on the spool is the sum of these components (21):

21 $$F_{HD}=-F_{HDS}-F_{HDT}=-\rho \frac{Q^2}{S_p}\cos(\theta)-\rho L \frac{\partial Q}{\partial t}$$

For the same spool stroke but opposite flow direction, the sign of the steady-state component remains unchanged, whereas the sign of the transient component reverses. The sign of the steady-state term only changes when the spool stroke reverses direction. Hydrodynamic forces can have a significant impact on the dynamic stability of the valve; therefore, they must be included in the mathematical model. Since the topic of hydrodynamic forces acting on the spool of a proportional valve has been addressed in detail in a previous study^41^, where the steady-state component was experimentally identified, in the present model the value of *F*_*HDS*_ is defined by a data matrix as a function of spool stroke *x* and volumetric flow rate *Q*. This matrix enables accurate interpolation of steady-state force values for any combination of *x* and *Q* within the experimental range. The transient component of the hydrodynamic force is determined according to Eq. (20), derived from Newton’s second law, which accounts for the time derivative of the flow rate $\frac{\partial Q}{\partial t}$. This modeling approach ensures that the simulation framework faithfully captures the influence of both components of the hydrodynamic force on the dynamic behavior of the valve.

## Simulation model

**Fig. 6:**
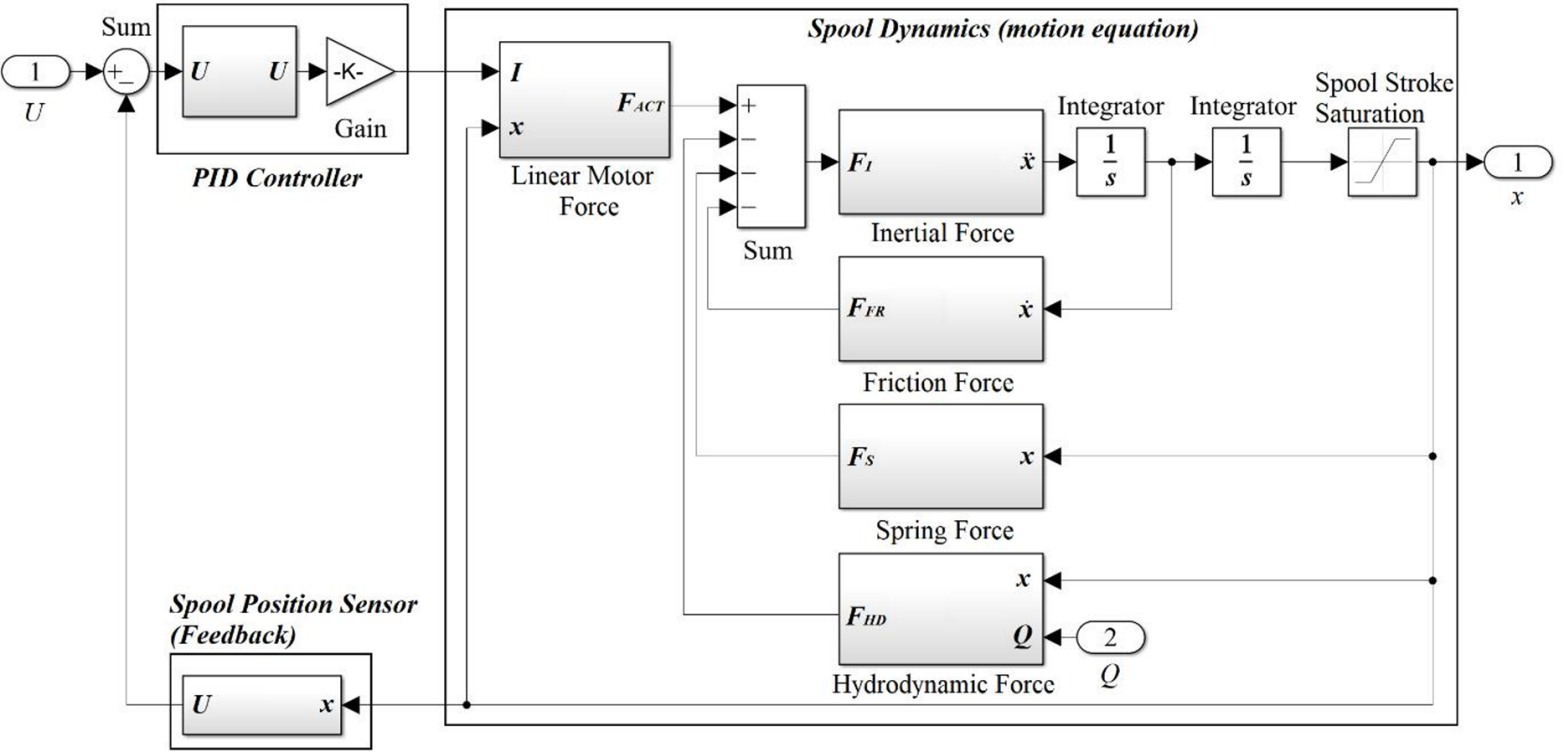
Structure of the model developed in Matlab Simulink.

Based on the previously identified parameters of the individual force components acting on the spool, a simulation model was developed in MATLAB/Simulink (see Fig. 6). The objective of this model is to simulate the dynamic behavior of the valve spool under various input and operating conditions. The model structure directly follows from the spool’s equation of motion, formulated earlier in the paper as Eq. (2). Each block in the model corresponds to a physical term in this equation, and their interconnections enable the numerical solution of the system dynamics. The developed model forms part of a comprehensive simulation model of the proportional valve, which incorporates both the spool dynamics and the hydraulic behavior of the working fluid flowing through the individual valve orifices^44^. However, within the scope of this study, only the mechanical part of the model (describing the spool motion itself) was employed for the identification of friction parameters and simulation of spool dynamics. The hydraulic subsystem was omitted to simplify the analysis. The output of the mechanical model is the spool stroke *x*, which subsequently serves as an input to the hydraulic part of the model. In this section, the flow rate *Q* is determined based on equations describing the fluid passage across the throttling edge, with the calculations accounting for the instantaneous spool position, pressure drop, discharge coefficient, and the density of the working fluid. The computed flow rate *Q* is then fed back into the mechanical model to evaluate the hydrodynamic force acting on the spool.

The model is driven by an input voltage signal *U*, which represents the desired spool stroke *x*. This signal is fed into a summation block, where it is combined with the negative feedback of the actual spool position converted into a corresponding voltage level. The difference between these two signals forms the control error, which is then processed by a PID controller. The controller parameters are provided in a subsequent section of the paper. The output of the controller is a voltage signal that is converted into a control current for the linear motor. This current, together with the actual spool position, determines the magnitude of the generated electromagnetic force, which constitutes the output of the linear motor force block. The generated force is subsequently fed into a second summation block, where it is combined with all forces opposing the spool motion. The resulting force balance corresponds to the spool’s equation of motion and, according to Newton’s second law, is equal to the product of the spool mass and its acceleration. Within the model, the spool acceleration is integrated to calculate its velocity and, subsequently, its position. The resulting spool position is constrained according to the physical stroke limits imposed by the valve design. This ensures a realistic representation of the mechanical boundaries of the system.

A key feature of the developed model is its modularity and high degree of parameterization. All critical parameters (such as stiffness, mass, friction coefficients, and geometric constraints) can be easily adjusted. As a result, the model is suitable not only for tuning and optimization but also for simulating open-loop operation (without feedback), thereby enabling comparisons of different control strategies and operating conditions.

The simulation model includes a PID controller that governs the spool position within a closed-loop control system. The controller is formulated in the parallel form with a filtered derivative term, and its transfer function in the Laplace domain is given by (22):

22 $$\:U\left(s\right)=P+\frac{I}{s}+D\frac{Ns}{s+N},$$

where *U(s)* denotes the transfer function of the controller, *s* is the Laplace variable, *P* is the proportional gain, *I* is the integral gain, *D* is the derivative gain, and *N* represents the filter coefficient of the derivative term^43^.

The proportional term provides an immediate response to the control error and primarily influences the response speed of the system. The integral term eliminates steady-state error by integrating the control error over time. The derivative term predicts the future trend of the error and improves damping; however, to avoid excessive amplification of high-frequency noise, it is implemented with a first-order low-pass filter defined by the parameter $\:N$.

Controller parameters were determined empirically by comparing simulated and experimental transient responses. The PID controller parameters were determined only after identifying the friction parameters of the moving assembly based on measurements performed under open-loop conditions. Their selection was guided by the objective of reproducing the dynamic behavior of the valve in its actual hardware configuration as accurately as possible, rather than by optimizing control performance. Since the controller parameters are neither specified by the manufacturer nor directly adjustable in the drive electronics, a manual tuning approach was adopted. The resulting controller parameters are as follows: *P* = 0.7, I = 350, D = 0. These values were determined within the MATLAB/Simulink environment. In the present study, the derivative gain was set to zero (D = 0), and the controller therefore operates as a PI controller. This configuration provides a sufficiently fast dynamic response while maintaining stability and minimizing overshoot. The absence of the derivative term eliminates sensitivity to measurement noise and contributes to stable performance even in the presence of minor nonlinearities or system variations.

## Experimental setup

For the purpose of performing an experimental analysis of the investigated proportional directional valve, a dedicated hydraulic test rig was designed and assembled. This setup enables measurements of both the static and dynamic characteristics of the tested proportional valve. To evaluate the dynamic behavior, both step-response (transient) and frequency-response characteristics were obtained, providing insight into the system’s time-domain response as well as its behavior across different frequency ranges. The experimental configuration also included a specially designed connection block, which allowed the installation of a force sensor for measuring the force characteristics of the valve. Using the same approach, it was also possible to measure the hydrodynamic forces acting on the spool.

### Test rig description

**Fig. 7:**
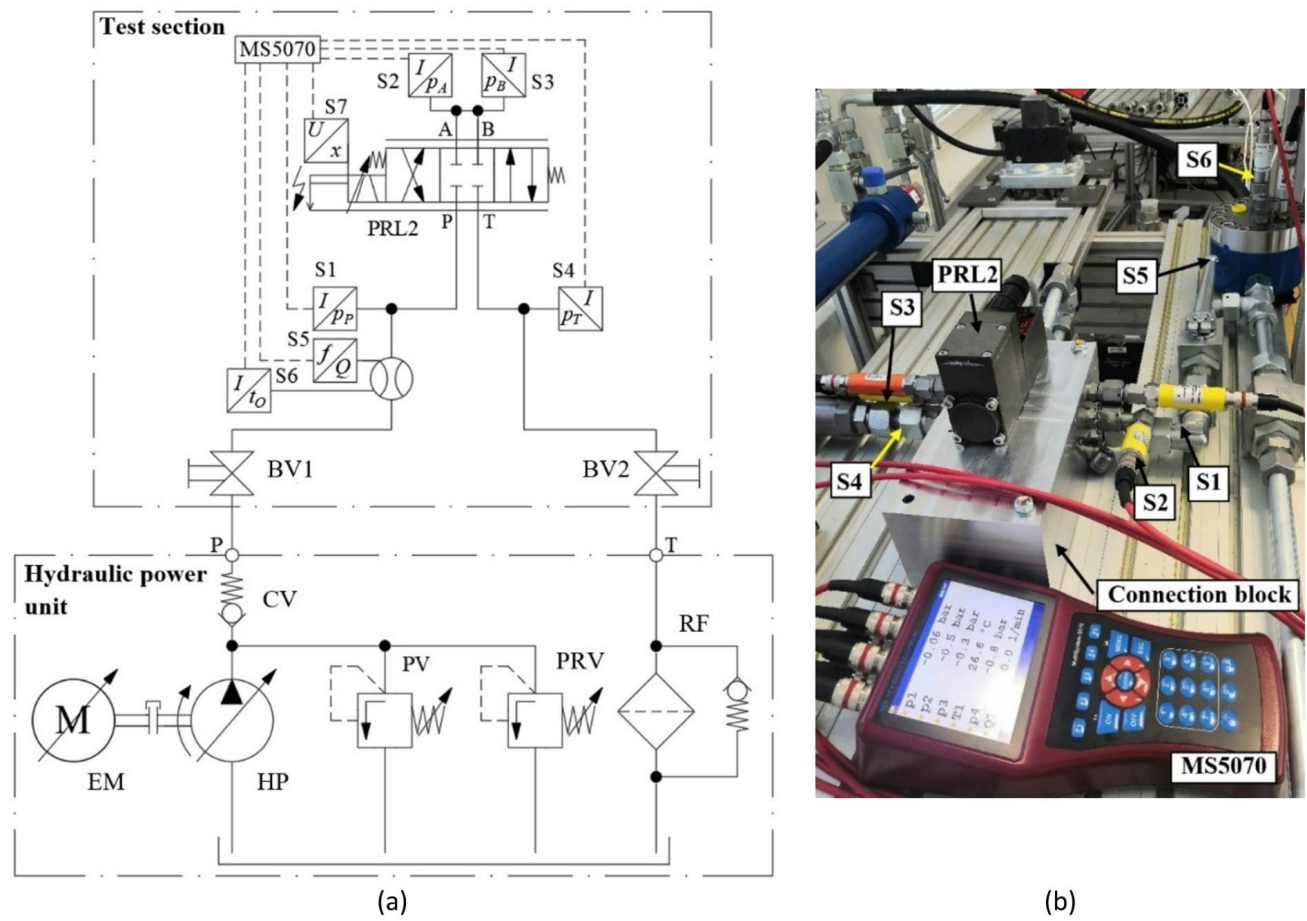
(**a**) Simplified hydraulic schematic of the test rig; (**b**) Test section of the rig with identification of measurement components^44^.

The experimental setup was designed with an emphasis on modularity and flexibility, allowing for easy adaptation to various types of measurements and configurations. The hydraulic circuit consists of two main sections: the hydraulic power unit and the test section. A simplified schematic of the entire hydraulic system is shown in Fig. 7a. The source of pressurized fluid is a hydraulic power unit comprising a swash-plate axial piston pump (HP) driven by an electric motor (EM). The rotational speed of the electric motor can be continuously controlled using a frequency converter. The maximum system pressure *p*_*PV*_ ​ is limited by a safety relief valve (PV), which also protects the entire hydraulic system against pressure overload. A pressure relief valve (PRV) is used to adjust and limit the working pressure *p*_*PPV*_ ​ in the hydraulic circuit.

The test section of the circuit is connected to the pressure line P and the return line T via ball valves BV1 and BV2. The investigated proportional directional valve PRL2 is mounted into the system using a specially designed hydraulic connection block that enables easy attachment of measuring devices and sensors. This block was designed to provide sufficient flexibility for supplementary measurements. Pressure sensors S1, S2, S3, and S4 are installed on the hydraulic block to measure the pressures *p*_*P*_, *p*_*A*_, *p*_*B*_ and *p*_*T*_ at the respective valve ports. The volumetric flow rate *Q* is measured by a gear flowmeter S5 located upstream of the valve. The hydraulic fluid temperature *t*_*O*_ is monitored using a temperature sensor S6 placed in a thermowell integrated into the flowmeter. The spool stroke *x* is measured by an integrated inductive position sensor S7, which is part of the proportional valve assembly. All sensors are connected to a Hydrotechnik MS5070 measuring device used for data acquisition and analysis. Figure 7b shows a detailed schematic of the test section with all key components and sensors highlighted. The specifications of the sensors, including their measurement ranges and accuracies, are summarized in Table 2.

**Table 2:** List of sensors used in the test rig.

Label	Sensor type	Measurement range	Accuracy
S1	Pressure sensor	(0–40) MPa	± 0.25% of full scale
S2, S3	Pressure sensor	(0–25) MPa	± 0.25% of full scale
S4	Pressure sensor	(0–6) MPa	± 0.25% of full scale
S5	Flow meter	(0.7–70) dm^3^⋅min^− 1^	up to ± 0.4% of reading
S6	Temperature sensor	(− 50–200) °C	0.3 + 0.005 *t*_*O*_ °C

### Measurement methodology

A series of measurements was conducted to determine the step responses and frequency responses of the investigated proportional directional valve. In this context, the step response is defined as the output variable (spool stroke) resulting from a step change in the control signal. The frequency response was obtained by evaluating the logarithmic amplitude and phase frequency characteristics. The input variable was the control voltage signal *U*, corresponding to the commanded spool stroke. The output variable was the actual spool stroke *x*, measured using the integrated inductive position sensor. Both variables were recorded with a sampling interval of 0.1 ms using the Hydrotechnik MS5070 measurement system.

For the experimental analysis of spool dynamics, five experimental variants were employed, as summarized in Table 3. The FB–nF variant, with feedback and no flow, was used to analyze the dynamic behavior of the valve and to identify the parameters of the PID controller, which were not known a priori. Measurements were intentionally conducted without fluid flow in order to eliminate the influence of hydrodynamic forces and to enable an unambiguous evaluation of the control loop behavior. In addition, two closed-loop configurations with fluid flow were investigated, namely FB–F–p5 and FB–F–p10, in which the measurements were performed with spool position feedback at different inlet pressures. These variants were used to evaluate the influence of fluid flow and inlet pressure on the friction force parameters and, consequently, on the dynamic behavior of the valve. The nFB–nF variant, without feedback and without flow, served for the identification of friction parameters. This configuration was intentionally selected as a reference identification state to eliminate the influence of hydrodynamic forces as well as the effects of feedback and the PID controller, thereby allowing isolation of the intrinsic mechanical friction between the spool and the valve body under controlled conditions. This variant was not intended to represent actual operating conditions but rather to provide a baseline for subsequent analyses. Finally, the nFB–F–p10 variant, without feedback but with fluid flow, was used to assess the influence of hydrodynamic forces acting on the spool and their effect on the valve dynamics.

**Table 3:** Overview of the experimental variants and their abbreviations.

FB–nF	Feedback No flow	Closed-loop feedback from the spool position with zero flow and zero system pressure, i.e., without hydrodynamic force acting on the spool
nFB–nF	No feedback No flow	Open-loop configuration without spool position feedback, with zero flow and zero system pressure. This setup is intended for the identification of friction parameters for the simulation model
FB–F–p5 FB–F–p10	Feedback flow	Closed-loop configuration with spool position feedback and fluid flow through the valve, resulting in hydrodynamic forces acting on the spool. These measurements were performed at inlet pressures of *p*_*p*_ = 10 MPa (FB–F–p5) and *p*_*p*_ = 10 MPa (FB–F–p10) to identify friction parameters under fluid flow conditions
nFB–F–p10	No feedback Flow	Open-loop configuration without spool position feedback, with fluid flow through the valve, resulting in hydrodynamic force acting on the spool. These measurements were performed at an inlet pressure of *p*_*p*_ = 10 MPa

During the measurements with fluid flow, in addition to voltage and spool stroke, the instantaneous pressures *p*_*P*_, *p*_*A*_, *p*_*B*_, *p*_*T*_ and the volumetric flow rate *Q* were recorded. The acquired data were evaluated using HYDROcom6 software and Microsoft Excel. The working fluid was HV46 hydraulic oil. The oil temperature during the individual measurements was maintained at *t*_*O*_ = 40 °C, corresponding to a kinematic viscosity of *ν* = 45.3 mm^2^⋅s^− 1^.

#### Step response tests

The step response of the proportional valve was experimentally determined for step changes of the control voltage *U* of 25%, 50%, 75% and 100%, where the maximum value corresponds to *U* = 10 V. This voltage simultaneously corresponds to the maximum spool stroke of *x* = 0.57 mm, which is considered as 100% of the total stroke. The step signals were generated using the MATLAB Simulink environment and applied to the proportional valve via the control electronics.

**Fig. 8:**
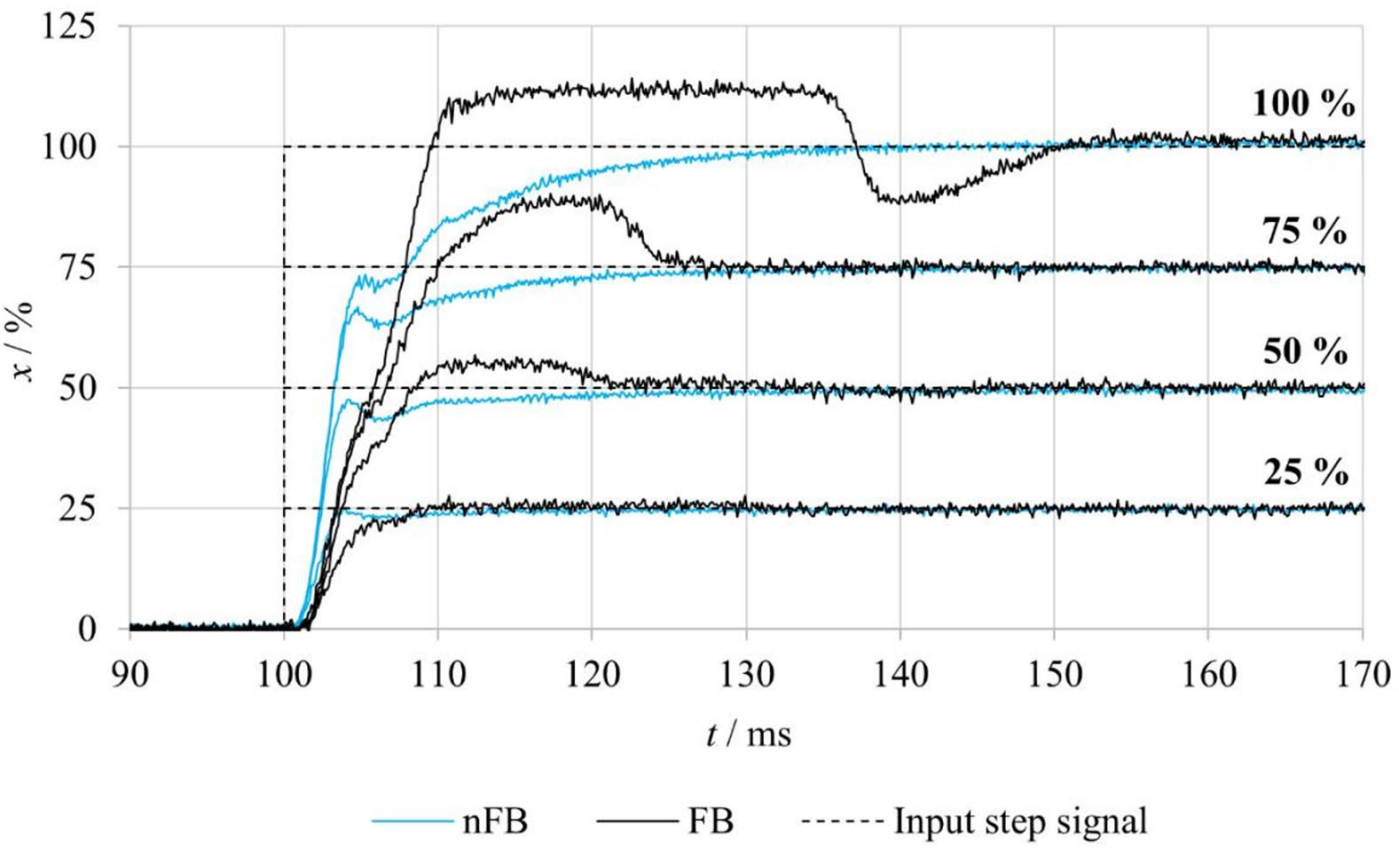
Comparison of step responses of the tested valve under FB–nF and nFB–nF conditions.

Figure 8 presents the evaluated step responses of the spool position for both **FB–nF** and **nFB–nF** experimental variants. In the **FB-nF** mode, the results indicate that the overshoot of the spool position increases with the magnitude of the input signal. At a 100% step change, the overshoot was constrained by the mechanical end stop of the spool, which was subsequently followed by a minor undershoot. Conversely, no overshoot was observed for the 25% input step. Furthermore, the settling time of the output response increases progressively with larger step amplitudes.

In the **nFB-nF** mode, the system operates under open-loop control, and the spool response represents solely the inherent dynamic properties of the actuator and the valve mechanics. Across all step input magnitudes, the output exhibited oscillatory behavior before gradually reaching the steady-state position. This phenomenon is attributed to the intrinsic dynamic behavior of the linear motor.

A comparison of the spool position step responses for the **FB–nF** and **nFB–nF** measurement variants reveals that, in the FB–nF mode, the spool position is controlled with higher accuracy and reduced oscillations, although the settling time increases slightly with larger input steps. At higher input levels, an overshoot is observed; in the case of the 100% input, this overshoot is constrained by the mechanical end stop of the spool and is followed by a minor undershoot. Conversely, in the **nFB–nF** mode, the initial rise of the spool position occurs more rapidly. The system exhibits an underdamped response, reflecting the absence of position feedback from the spool position. These differences in dynamic behavior clearly demonstrate the crucial role of feedback in suppressing oscillations and facilitating faster attainment of the desired position.

**Fig. 9:**
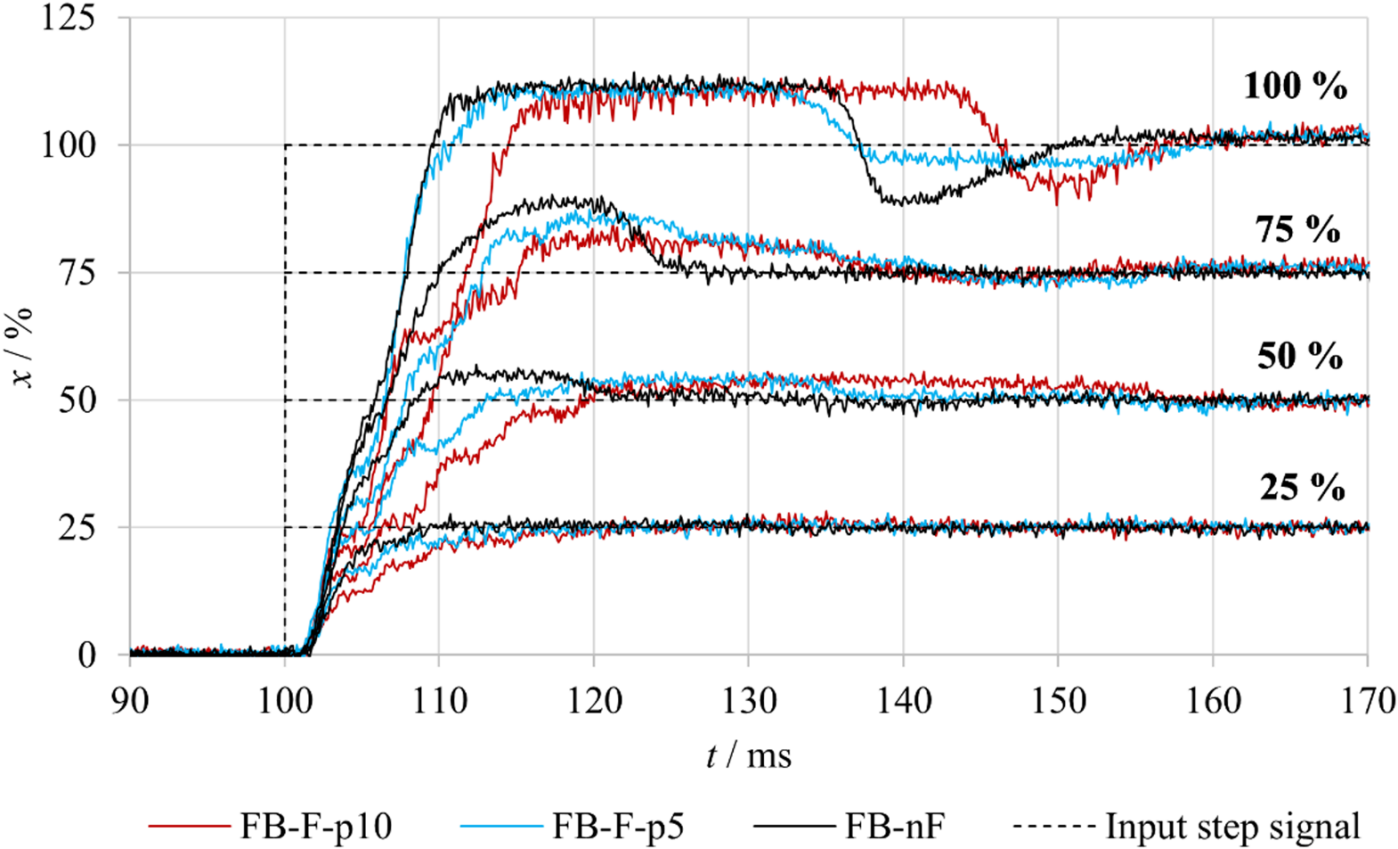
Comparison of step responses of the tested valve under FB–nF and FB–F conditions.

Figure 9 compares the spool position responses to a step change in the input signal for the **FB–nF** and **FB–F** configurations. In the case of fluid flow, the measurements were performed at two different inlet pressures. Comparison of the responses for identical input signals shows that the spool position response becomes progressively slower with increasing inlet pressure, as indicated by an increased rise time and settling time. Despite the altered dynamic behavior, the steady-state spool position remains largely unaffected.

Under flow conditions, hydrodynamic forces act on the spool and modify the loading and friction conditions between the spool and the valve body, thereby influencing the dynamic response of the system. This behavior was consistently observed across all tested input signal levels, indicating a systematic effect of fluid flow on the valve dynamics.

**Fig. 10:**
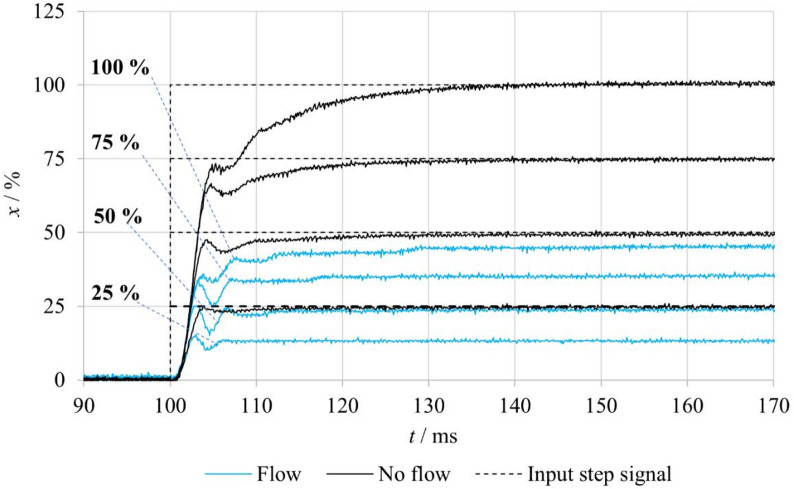
Comparison of step responses of the tested valve under nFB–nF and nFB–F conditions.

Figure 10 compares the spool position step responses to input signal changes in the **nFB–nF** and **nFB–F** modes. In the absence of fluid flow, the spool position exhibits oscillatory behavior before stabilizing at the value corresponding to the input command. When fluid flow is present, the oscillation amplitude increases, and a steady-state deviation of the actual spool position from the desired one emerges, with the magnitude of this deviation becoming more pronounced at higher input levels. This effect results from the hydrodynamic force acting in the closing direction of the flow channels, which remains uncompensated in the open-loop configuration. The findings highlight the substantial influence of hydrodynamic forces on the spool’s dynamic response and emphasize the necessity of accounting for them in both experimental evaluation and the development of mathematical models of the valve.

Quantitative parameters of the step responses, such as the overshoot magnitude, rise time, and settling time, are summarized and compared in Table 4. The overshoot represents the extent to which the actual spool displacement exceeds the desired position, the rise time defines the duration required for the spool to reach the target value (without necessarily stabilizing there), and the settling time corresponds to the time needed for the spool position to stabilize within the desired value.

**Table 4:** Parameters of the step responses.

Mode	Parameter	25%	50%	75%	100%
FB–nF	Overshoot/%	–	5.2	14.1	11.7
	Rise time/ms	8.7	8.4	10.1	9.5
	Settling time/ms	8.7	20.7	23.4	47
FB–F–p5	Overshoot/%	–	3.9	11.1	11.7
	Rise time/ms	16.1	16.5	12.5	10.8
	Settling time/ms	16.1	36.4	39.8	56.1
FB–F–p10	Overshoot/%	–	3.8	8.1	11.7
	Rise time/ms	20.9	19.1	15.1	14
	Settling time/ms	20.9	55.9	37.6	55
nFB–nF	Overshoot/%	–	–	–	–
	Rise time/ms	4.1	24.5	25.7	32.1
	Settling time/ms	11.2	24.5	25.7	32.1
nFB–F–p10	Overshoot/%	5	5.9	–	–
	Rise time/ms	2.6	2.6	26.9	28
	Settling time/ms	6	11.4	26.9	28

#### Frequency response tests

The frequency responses of the investigated proportional valve were experimentally determined based on harmonic excitation in the frequency range *f* = (0–100) Hz. The input signal was a harmonic waveform with a mean value (offset) of *U* = 0 V and an amplitude of *U* = ± 2.5 V, corresponding to 25% of the maximum control voltage. The signals were generated in MATLAB Simulink, which enables precise specification of the frequency profile, including amplitude and offset. To evaluate the amplitude frequency response, the system attenuation was expressed as the logarithmic attenuation *L* in decibels according to Eq. (23):

23 $$\:L=20\cdot\:\mathrm{log}\frac{{A}_{x}}{{A}_{U}},$$

where *A*_*x*_*/A*_*U*_ represents the amplitude ratio, expressed as a percentage, between the spool stroke *x* and the input voltage signal *U*^45^. This method enables a quantitative evaluation of the system’s frequency response, capturing both gain attenuation and phase variations as a function of the excitation frequency.

**Fig. 11:**
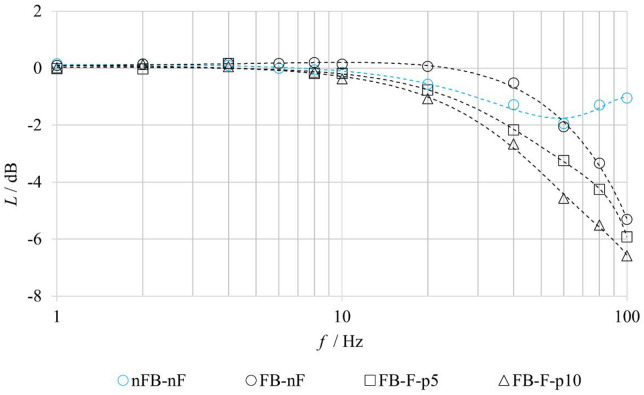
Frequency response magnitude of the tested valve under FB–nF, FB–F and nFB–nF conditions.

Figure 11 presents the logarithmic amplitude frequency responses $\:L$ as a function of the harmonic excitation frequency *f*, evaluated for the **FB–nF**, **FB–F**, and **nFB–nF** configurations. The frequency response of the valve with feedback shows that at lower frequencies (approximately up to 20 Hz), the system exhibits a slight amplification of the output amplitude, where the amplitude ratio *A*_*X*_/*A*_*U*_ >1, corresponding to a positive attenuation value *L* (commonly referred to as amplitude overshoot). This behavior is characteristic of control loops containing dynamic elements, in which feedback enhances the system response to the excitation signal. At higher frequencies, above approximately 20 Hz, the response amplitude decreases, indicating the limited frequency bandwidth of the system. In contrast, measurements performed without feedback exhibit no overshoot; the response amplitude decreases monotonically with increasing frequency up to approximately 60 Hz, reflecting the lower dynamic sensitivity and more passive behavior of the open-loop system. For frequencies above approximately 60 Hz, a reduction in amplitude attenuation is observed for the configuration without feedback, which can be attributed to the mechanical natural frequencies of lightweight components with insufficient hydraulic damping. This phenomenon is not observed in configurations with feedback. A comparison of the frequency responses obtained with feedback under no-flow and flow conditions indicates that the amplitude attenuation increases with increasing pressure, and thus with increasing flow rate. For proportional valves, an amplitude attenuation of *L* = − 3 dB is commonly considered the upper limit of the usable frequency bandwidth. The evaluated frequency responses suggest an operational frequency range of the valve under flow conditions of approximately 40–50 Hz. These results highlight the critical role of feedback in extending the dynamic range and accelerating the response of the proportional valve.

**Fig. 12:**
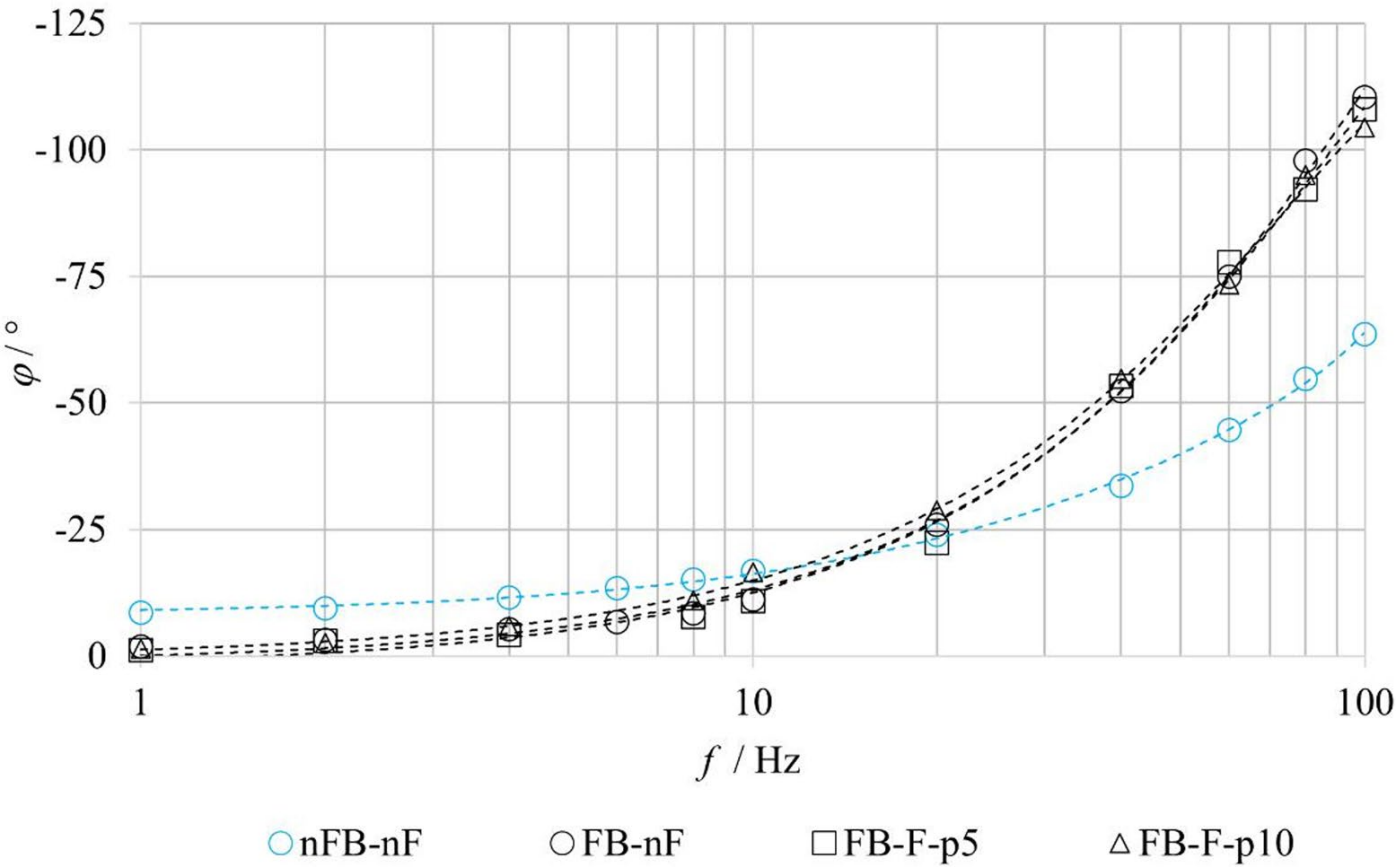
Phase response of the tested valve under FB–nF, FB–F and nFB–nF conditions.

Figure 12 presents the phase frequency responses of the valve, expressed as the phase shift *φ* between the input voltage signal and the spool output response as a function of excitation frequency *f*. All configurations exhibit a monotonic increase in phase shift with increasing frequency, which is characteristic of systems with dynamic delay. When comparing the **FB–nF** and **nFB–nF** configurations, it is evident that at lower frequencies (up to approximately 10 Hz), the feedback-enabled system exhibits a smaller phase shift, indicating a faster response to input excitation. At higher frequencies, however, feedback contributes to an increased dynamic phase shift, as the control loop introduces additional dynamics into the system transfer behavior. The more pronounced increase in phase shift for the feedback configuration at frequencies above approximately 20 Hz reflects the increasing difficulty of compensating the control error at higher excitation frequencies. A comparison of the **FB–nF** and **FB–F** configurations shows that the phase shift remains largely unaffected by increasing inlet pressure. These observations confirm the dynamic nature of the system and highlight the benefit of feedback, particularly in the low-frequency range, where it enables more accurate and faster tracking of the reference signal.

## Identification of friction model parameters

The identification of the friction force parameters *F*_*FR*_ was performed through a combined numerical and experimental analysis of the system’s response to step control signals. Initially, a comprehensive simulation model of the spool motion was developed in MATLAB Simulink, incorporating all known forces acting on the spool, including the linear motor force, inertial force, and spring forces. These components were pre-determined based on the experimental measurements presented in previous sections and were fixed in the model. The experiments were conducted under zero-flow conditions, with the valve fully filled with hydraulic oil, including the valve chambers and the radial clearance between the spool and the valve body. In this configuration, the oil did not flow and no pressure gradient was applied, thereby eliminating the influence of hydrodynamic forces on the spool and allowing the identification of the inherent mechanical friction of the moving assembly. Furthermore, the experiments were carried out with the spool position feedback disabled (**nFB–nF**), removing the influence of the valve control electronics. Subsequent experiments performed under flow conditions and different inlet pressures demonstrated that the effective friction characteristics vary with operating conditions. These effects are therefore addressed separately in the following section, where the influence of fluid flow and inlet pressure on the identified friction parameters is analyzed.

With this simulation environment established, the friction force model *F*_*FR*_ described by Eqs. (16)–(18) was tuned. The model incorporates a nonlinear friction representation corresponding to the Stribeck effect, including the Coulomb component, viscous damping, and a velocity-dependent term capturing the transition between static and dynamic friction. The individual friction parameters (namely the Coulomb friction force *F*_*C*_, breakaway friction force *F*_*ST*_, viscous friction coefficient *f*_*V*_ and breakaway friction velocity *v*_*BR*_) were adjusted sequentially, and their influence on the resulting spool motion was systematically evaluated.

**Fig. 13:**
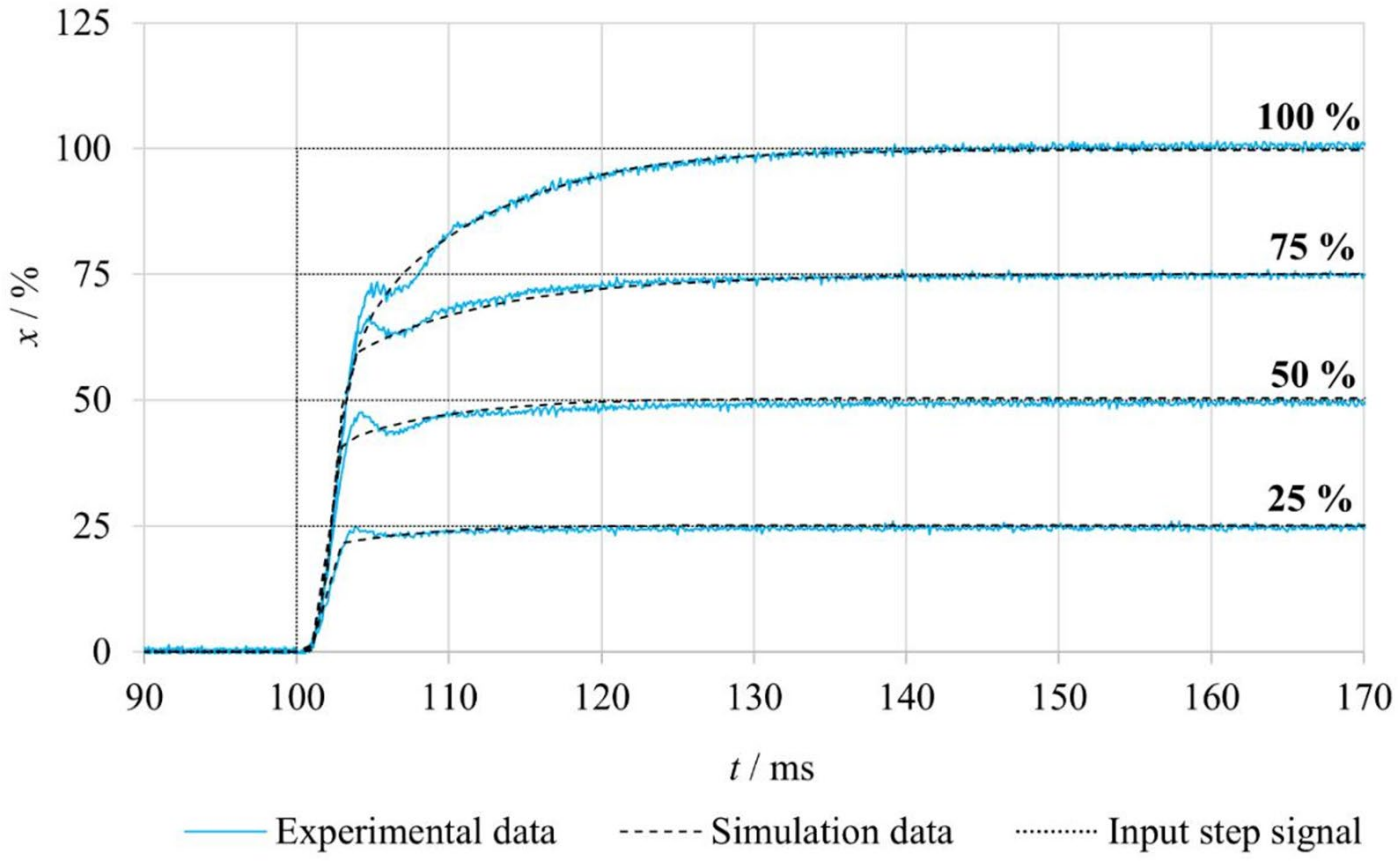
Comparison of experimental and simulated spool position responses for different step input signals, measured under nFB–nF conditions.

The parameters of the friction force model *F*_*FR*_, presented in Table 5, were identified through a comparison of experimentally measured and simulated dynamic spool responses. Identification was conducted for varying step amplitudes of the control voltage *U*, with the primary outputs being the breakaway friction force *F*_*ST*_, breakaway friction velocity *v*_*BR*_, Coulomb friction force *F*_*C*_ and viscous friction coefficient *f*_*V*_. To facilitate comparison between open-loop and closed-loop configurations, step voltages were chosen such that the steady-state spool positions corresponded to 25%, 50%, 75%, and 100% of the total stroke, consistent with the feedback configuration. The results indicate that *F*_*ST*_ increases with input amplitude, while the other parameters remain constant. According to the implemented friction model, the breakaway friction force is defined as the difference between static and Coulomb friction, which determines the magnitude of the first term in the governing equation. The breakaway friction force must always be greater than or equal to the Coulomb friction force to correctly capture the transition between static and dynamic friction. Higher voltage excitations of the linear actuator induce a faster and more abrupt spool acceleration, which, combined with adhesive effects at contact surfaces, leads to an effective increase in *F*_*ST*_. Figure 13 illustrates the time-dependent spool position *x*(*t*) for the various step input signal amplitudes *U*, showing both experimental and simulated data for each control input. The simulation model incorporating the friction model demonstrates close agreement with the measured data across the entire tested range. Minor discrepancies are observed during the initial motion phase. They are likely caused by transient effects when overcoming static friction or by small nonlinearities that are not captured by the model. Overall, the findings confirm a high level of fidelity between the physics-based simulation and experimental results.

**Table 5:** Parameters of the friction force *F*_*FR*_ for different step input signals.

x/%	F_ST_/*N*	v_BR_/m⋅s^− 1^	F_C_/*N*	f_V_/*N*⋅s⋅m^− 1^
100	35	0.16	10	20
75	18.7			
50	13.5			
25	4.25			

To increase the validity of the friction parameter identification, the simulation model was further verified based on the system’s frequency response. Experimentally and numerically determined frequency characteristics were compared for the open-loop configuration without spool position feedback and without fluid flow through the valve (**nFB–nF**). A harmonic input signal was applied, with an amplitude corresponding to 25% of the maximum spool stroke and an offset set to the mean spool position. Simulations using friction parameters identified from the step response corresponding to the 25% spool stroke level (see Table 5) revealed significant discrepancies between the numerically predicted and experimentally measured frequency responses. This demonstrated the necessity of a dedicated friction parameter identification specifically for harmonic excitation.

Within this identification, it was found that the breakaway friction force *F*_*ST*_ and the Coulomb friction force *F*_*C*_ remained constant even under harmonic spool motion. In contrast, the breakaway friction velocity was identified as *v*_*BR*_ = 0.35 m⋅s^− 1^ while the viscous friction coefficient exhibited a value of *f*_*V*_ = 18 N·s·m⁻¹. The variation in these parameters may be attributed, for example, to the enhanced influence of dynamic resistance at higher mean spool velocities in the harmonic regime.

**Fig. 14:**
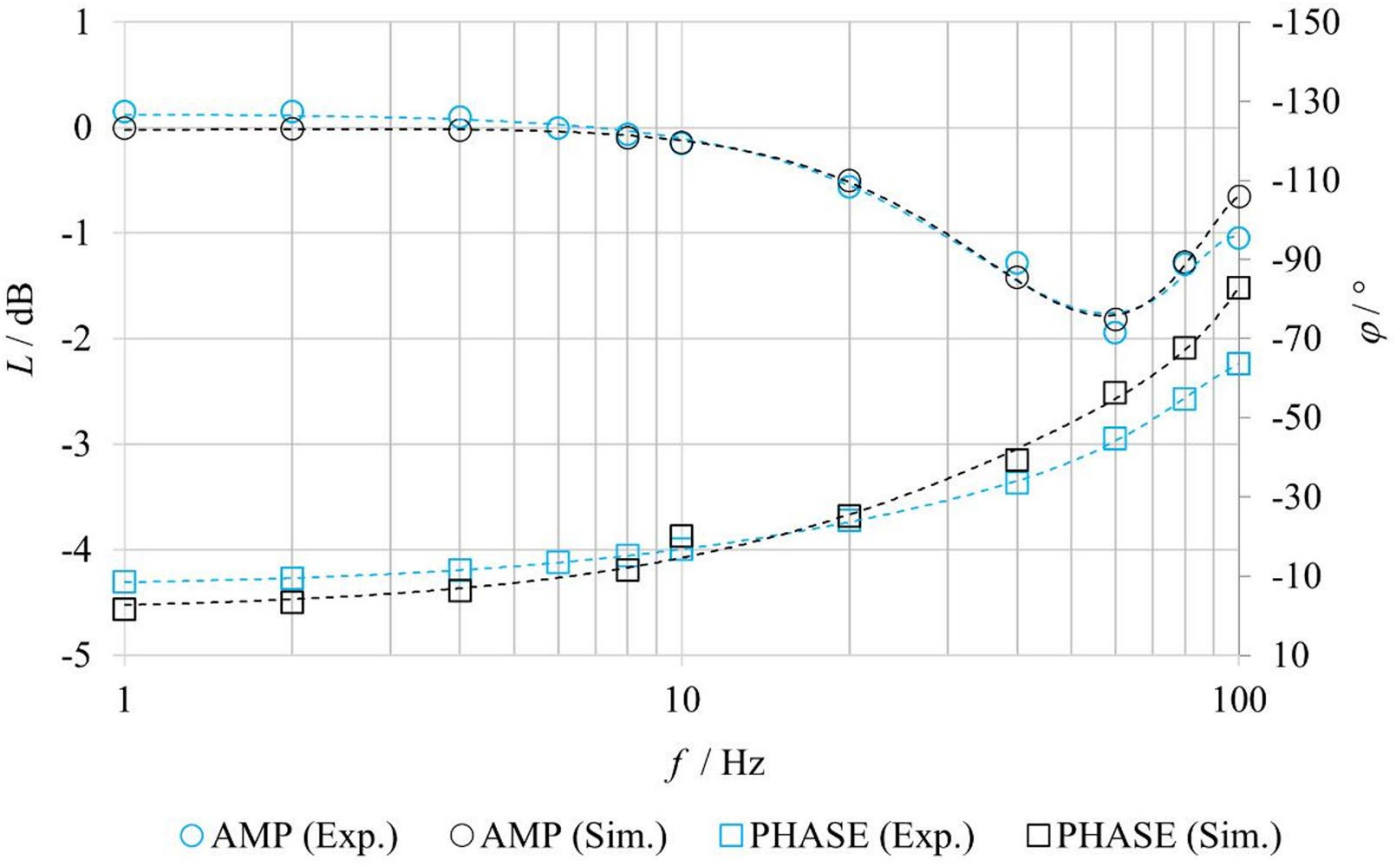
Comparison of experimentally and numerically obtained logarithmic magnitude and phase frequency responses under nFB–nF conditions.

Figure 14 presents the logarithmic frequency characteristics (amplitude and phase) of the valve, obtained both experimentally and through simulation with the newly identified friction parameters. The results indicate that the simulated model reproduces the amplitude response of the system with high accuracy across the entire frequency range. The difference between measured and simulated amplitudes is practically negligible. The model also accurately captures the resonant region of the system, where an increase in amplitude occurs, and correctly reproduces the location of the resonant frequency. In contrast, the phase characteristic exhibits a slight deviation, which increases with frequency. This deviation may be attributed to nonlinearities in the motion system, that are not fully captured by the selected friction model, or to dynamic phenomena arising from the interaction between the mechanical and electrical subsystems of the valve. Despite this minor discrepancy, the model demonstrates close agreement with the experimental data in the harmonic regime and confirms its ability to reliably reflect the system dynamics.

## Results and discussion

In this chapter, the step responses and frequency characteristics of the system in a closed-loop configuration without fluid flow (FB–nF) are first compared. The friction parameters identified in the previous chapter under the nFB–nF condition are employed in the numerical model to perform simulations for the FB–nF configuration. Subsequently, the step responses and frequency characteristics of the system in a closed-loop configuration with fluid flow (**FB–F**) are analyzed and compared. For this comparison, modified friction parameters are employed, as fluid flow influences the friction conditions within the valve. The step responses and frequency characteristics of the system in an open-loop configuration with fluid flow (**nFB–F**) are then analyzed and compared. The chapter concludes with a comparative analysis of the proposed physics-based model and a commonly used linear model in the form of a second-order transfer function, aimed at assessing the benefits of the increased model complexity under operating conditions with fluid flow.

**Fig. 15:**
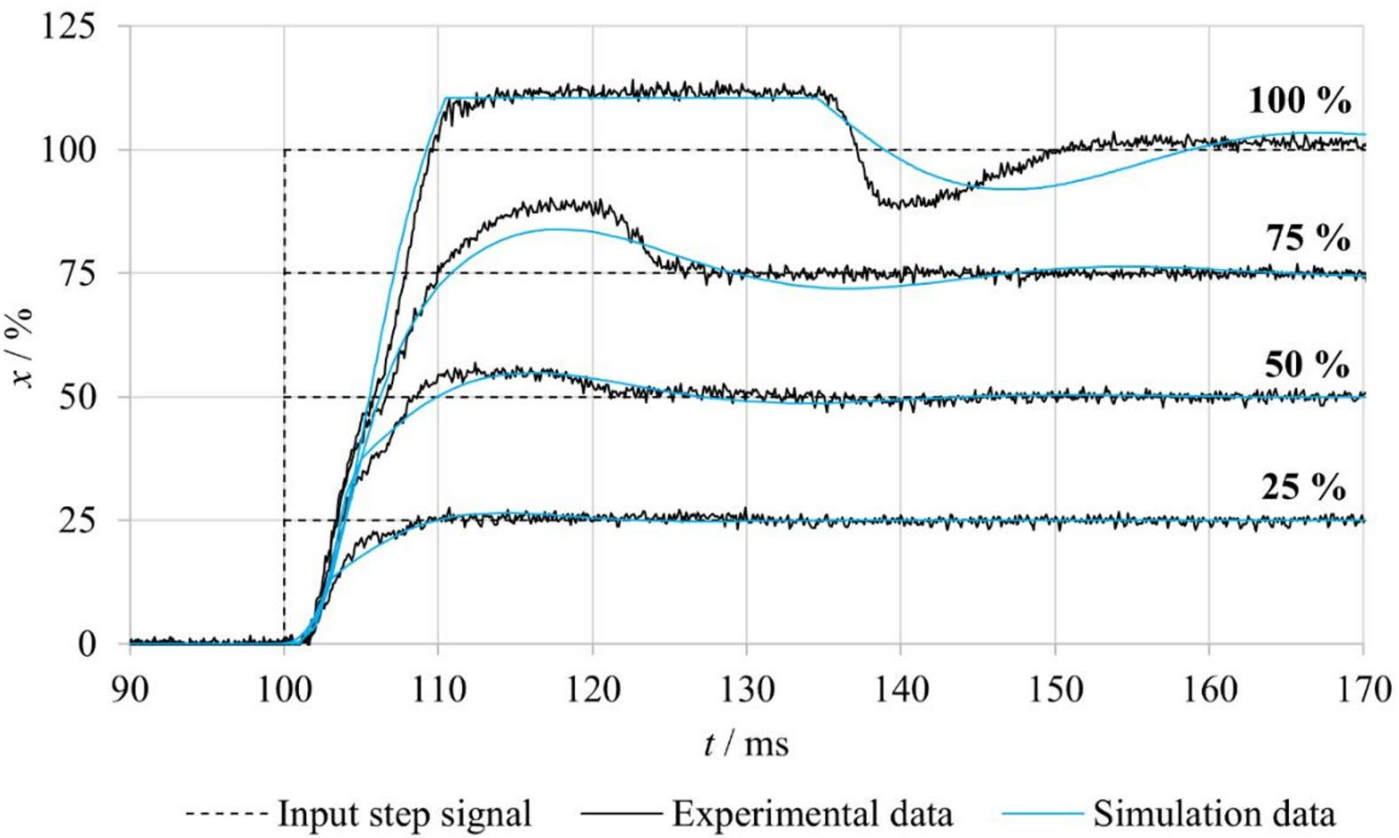
Comparison of measured and simulated spool position responses for different step input signals under FB–nF conditions.

Figure 15 compares the experimentally measured and numerically predicted spool position responses for step input signals of 25%, 50%, 75%, and 100%, presented here for the **FB–nF** mode. The results show that the simulation model captures the response very accurately for lower input amplitudes (25% and 50%), with only minimal differences observed in rise time and steady-state values. At higher input levels (75% and 100%), larger discrepancies become evident, particularly immediately after the commanded value is reached. The experimental responses exhibit slight deviations that are not fully reproduced by the simulations. These discrepancies may arise from nonlinearities or unmodeled effects, such as nonlinear characteristics of the control elements or time-, temperature-, and load-dependent variations in friction forces.

**Fig. 16:**
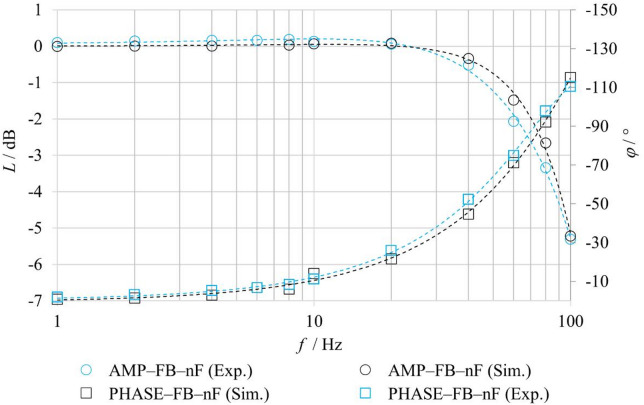
Comparison of experimentally and numerically obtained logarithmic magnitude and phase frequency responses under FB–nF conditions.

Figure 16 compares the experimentally measured and numerically predicted frequency characteristics of the system in the **FB–nF** configuration. The plot includes the amplitude characteristic, expressed as the amplitude attenuation *L* and the phase characteristic, expressed as the phase shift *φ*, both as functions of the excitation frequency *f*, with the frequency axis shown on a logarithmic scale. The simulation model reproduces the system behavior very well, showing good agreement with experimental data across the investigated frequency range. At lower frequencies, the simulated amplitude characteristic exhibits only a slight decrease, whereas the experimental response shows a minor increase in amplitude. For frequencies above approximately 10 Hz, the simulated amplitude remains slightly higher than the experimentally measured values. The comparison of amplitude characteristics confirms that the model correctly captures the overall attenuation *L* as a function of frequency *f*. For the phase characteristic, the agreement between simulation and experiment is likewise good, with only small deviations observed at intermediate frequencies. In these regions, the experimental data exhibit somewhat larger phase shifts, which may be attributed to delays in the real system, nonlinearities in the control loop, or external disturbances such as noise or mechanical resonances not accounted for in the model.

For the comparison of experimentally measured and numerically simulated spool position responses in the closed-loop configuration with fluid flow (**FB–F**), it was necessary to adjust the friction parameters used in the simulation model, as fluid flow influences the friction conditions within the valve. The identification of the modified friction parameters was carried out using the same procedure as described in the previous chapter. The identified friction parameters for individual step input amplitudes and different inlet pressures are summarized in Table 6. The results indicate that the static friction component *F*_*ST*_ increases with both increasing input signal amplitude and increasing inlet pressure, with the highest values observed for the FB–F–p10 configuration. A similar, although less pronounced, trend is also evident for the Coulomb friction component *F*_*C*_. In contrast, the viscous friction coefficient *f*_*V*_ remains constant for all considered configurations, suggesting that the viscous friction component is relatively insensitive to variations in inlet pressure and fluid flow under the investigated conditions. The increase in the identified Stribeck velocity *v*_*BR*_ under flow conditions further indicates modified lubrication and contact conditions between the spool and the valve body.

**Fig. 17:**
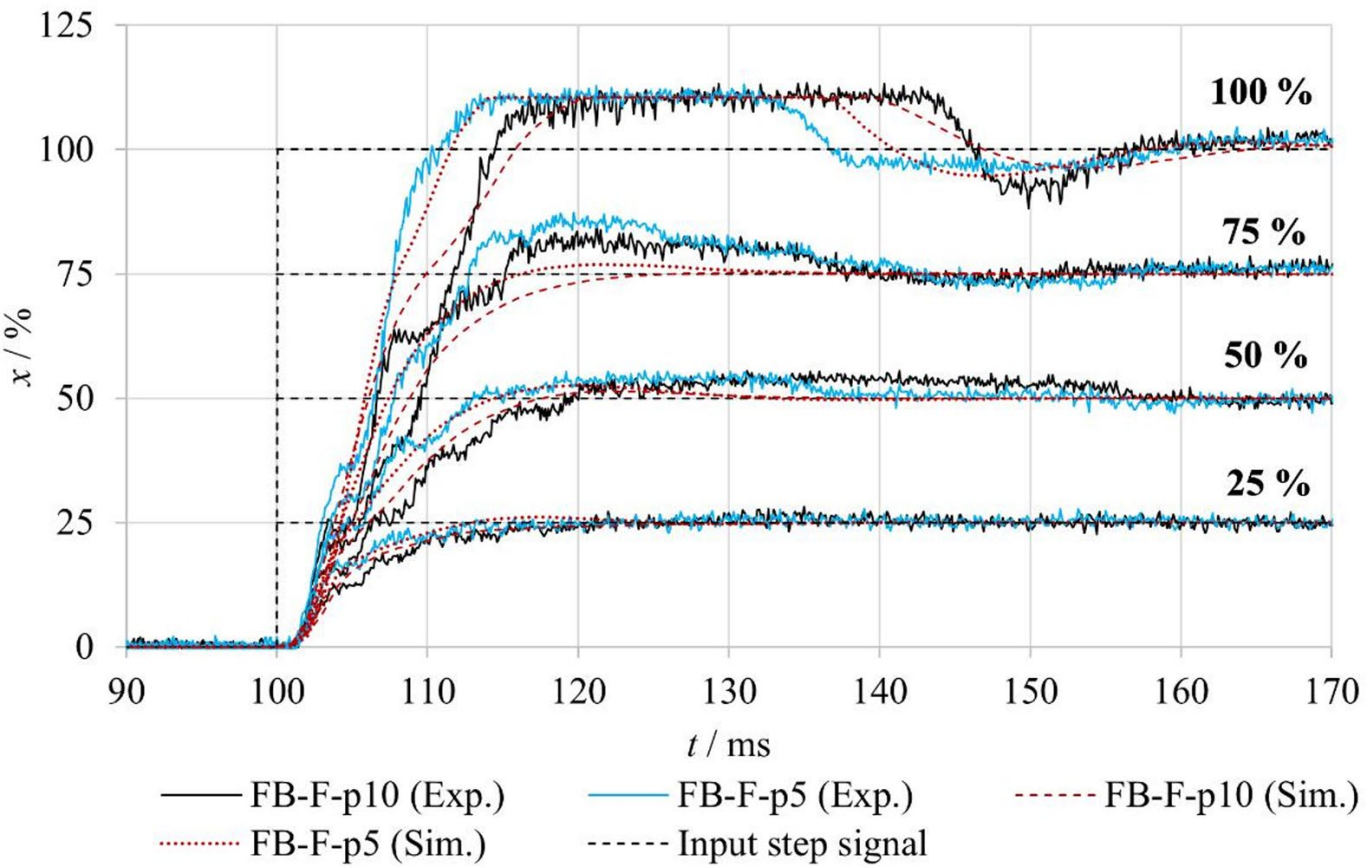
Comparison of measured and simulated spool position responses for different step input signals under FB–F conditions.

Based on these adjusted friction parameters, numerical simulations were performed and compared with the experimental data, as shown in Fig. 17. The comparison demonstrates that the simulation model accurately reproduces the spool position rise behavior and the steady-state values for all input signal levels. However, certain discrepancies between the simulation and the experiment are observed in the overshoot region during the transient response. These differences can be attributed to dynamic and nonlinear properties of the real control electronics that are not explicitly included in the numerical model. Despite these deviations, the achieved agreement confirms that the proposed model is capable of reliably capturing the influence of fluid flow and friction on the dynamic behavior of the spool motion.

**Table 6:** Parameters of the friction force *F*_*FR*_ for different step input signals under no-flow and flow conditions.

x/%	Mode	F_ST_/*N*	v_BR_/m⋅s^− 1^	F_C_/*N*	f_V_/*N*⋅s⋅m^− 1^
25%	FB–nF	4.25	0.16	10	20
	FB–F–p5	6	0.16	11	
	FB–F–p10	8	0.16	12	
50%	FB–nF	13.5	0.16	10	
	FB–F–p5	20	0.2	11	
	FB–F–p10	25	0.2	12	
75%	FB–nF	18.7	0.16	10	
	FB–F–p5	25	0.2	11	
	FB–F–p10	30	0.2	12	
100%	FB–nF	35	0.16	10	
	FB–F–p5	43	0.2	11	
	FB–F–p10	50	0.2	12	

**Fig. 18:**
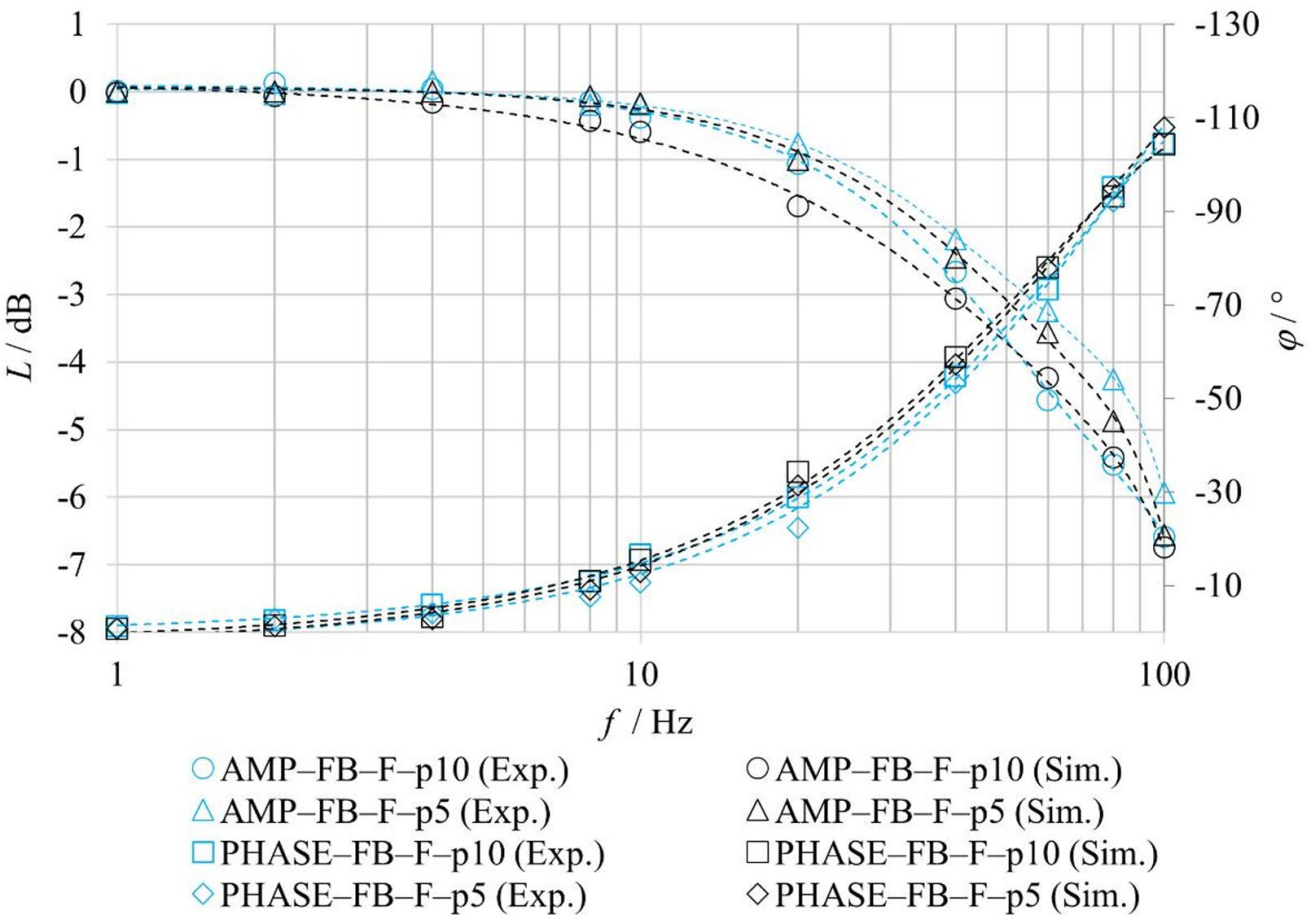
Comparison of experimentally and numerically obtained logarithmic magnitude and phase frequency responses under FB–F conditions.

For the comparison of experimentally measured and numerically simulated frequency characteristics in the **FB–F** configurations, the friction parameters of the model were also adjusted for the harmonic regime. Table 7 summarizes the identified friction parameters used in the simulation of the frequency characteristics for an excitation amplitude of ± 25% for the FB–nF and FB–F configurations. The results indicate that even under harmonic excitation, fluid flow leads to changes in the effective friction conditions within the valve. In particular, the static friction component *F*_*ST*_ and the Stribeck velocity *v*_*BR*_ exhibit higher values under flow conditions compared to the no-flow configuration, with the highest values observed at higher inlet pressure. The Coulomb friction component *F*_*C*_ exhibits lower values with increasing pressure, while the viscous friction coefficient *f*_*V*_ remains constant for all considered configurations. These parameters were subsequently used in the numerical calculation of the frequency characteristics shown in Fig. 18, and the achieved agreement between simulation and experiment confirms the necessity of accounting for the influence of fluid flow on friction conditions also in the harmonic regime.

**Table 7:** Parameters of the friction force *F*_*FR*_ for harmonic signal.

Mode	F_ST_/*N*	v_BR_/m⋅s^− 1^	F_C_/*N*	f_V_/*N*⋅s⋅m^− 1^
FB–nF	4,25	0,35	10	18
FB–F–p5	8	0,4	8	
FB–F–p10	10	0,4	9	

**Fig. 19:**
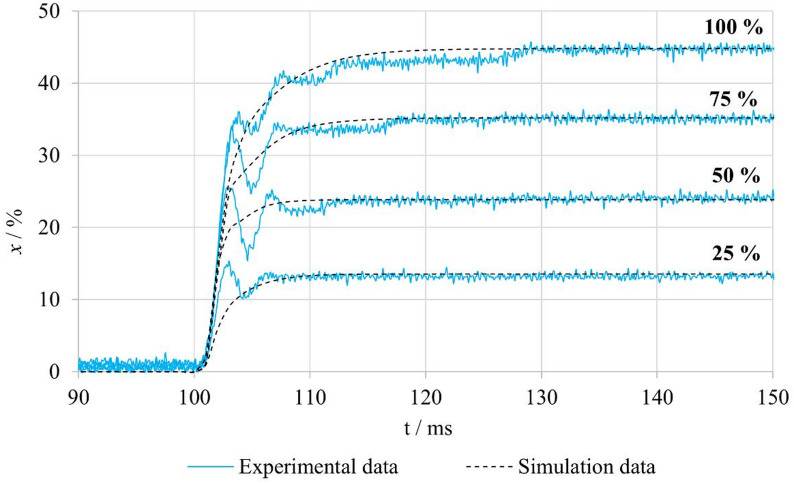
Comparison of experimental and simulated spool position responses for different step input signals under nFB–F–p10 conditions.

Figure 19 compares the experimentally measured and numerically predicted step responses of the system in the **nFB–F–p10** configuration, where fluid flow occurs and hydrodynamic forces act on the spool. The graph presents responses for four different amplitudes of the step input signal. The numerical simulations were performed using the friction parameters corresponding to this configuration, which were identified based on the experimental measurements and are summarized in Table 6. The measured responses demonstrate that hydrodynamic forces significantly affect system behavior, particularly during the initial phase of spool motion, where discrepancies between experiment and simulation become evident. In this phase, the model does not reproduce the oscillations or the associated short-term deceleration observed in the experiments. This phenomenon is likely related to the influence of fluid flow on the spool, which manifests more prominently in the real system. Although the model does not capture these oscillations, it accurately describes the steady-state values and the fundamental system dynamics. Further refinement of the model could be achieved by improving the parameterization of hydrodynamic forces or by extending the formulation to include nonlinear elements that describe the interaction between fluid flow and spool motion, especially at higher flow rates.

The overall comparison of results demonstrates that the proposed simulation model provides an accurate representation of the dynamic behavior of the proportional valve in both feedback and no-feedback configurations. For low to medium step input amplitudes and within the amplitude frequency response range, the simulation and experimental results show close agreement. Deviations become more pronounced at higher input levels and in the presence of hydrodynamic forces, where the real system exhibits more complex dynamics due to nonlinearities and interactions between fluid flow and mechanical components. The model therefore represents a suitable tool for predicting system behavior across a wide range of operating conditions, while the identified discrepancies indicate directions for further refinement, particularly in terms of more accurate modeling of variable friction phenomena and hydrodynamic forces.

**Fig. 20:**
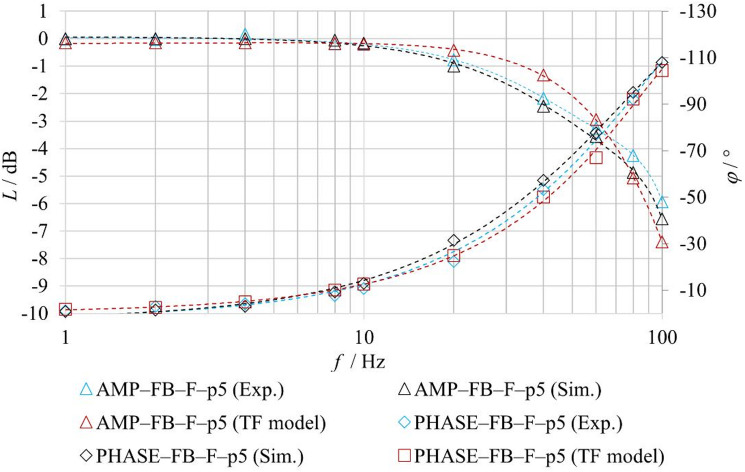
Comparison of experimentally measured and simulated amplitude frequency responses under FB–F–p5 conditions.

**Fig. 21:**
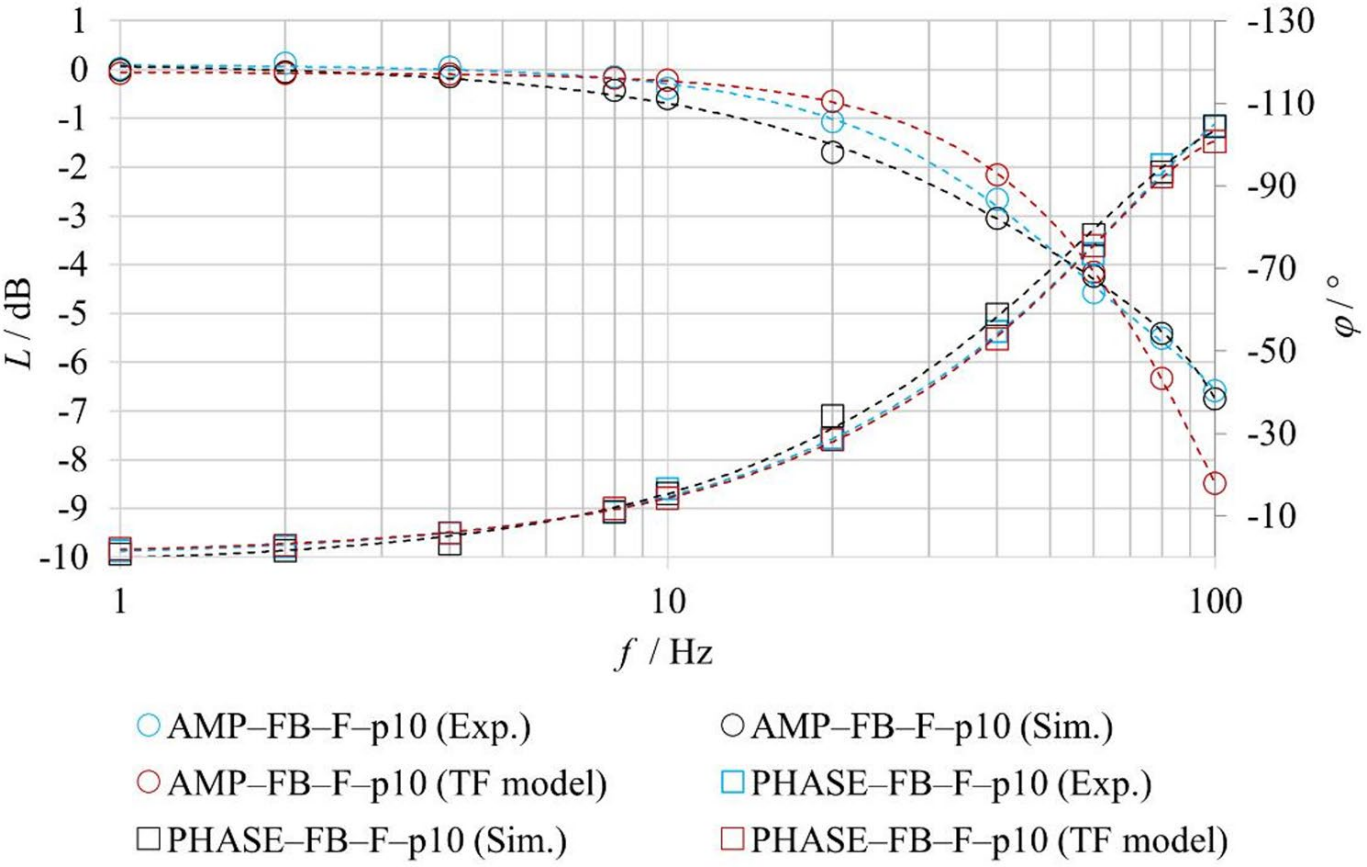
Comparison of experimentally measured and simulated amplitude frequency responses under FB–F–p10 conditions.

To evaluate the benefits of the proposed physics-based model, a comparison was performed with a conventional second-order transfer-function model. The analysis was conducted for the **FB–F–p5** and **FB–F–p10** configurations, under closed-loop operation with fluid flow. The transfer-function model, representing a second-order inertial system, corresponds to the formulation given in Eq. (1). The model parameters were identified from experimentally measured amplitude and phase frequency responses. The agreement between the experiment (Exp.), the physics-based model (Sim.), and the transfer-function model (TF model) was evaluated using amplitude and phase characteristics, as shown in Figs. 20 and 21.

Quantitative agreement was assessed using the MAE (Mean Absolute Error) and RMSE (Root Mean Square Error) metrics for amplitude (dB) and phase (deg) responses. The results are summarized in Table 8.

**Table 8:** Quantitative comparison of frequency response errors for the physics-based and transfer-function models under FB–F conditions.

		FB-F-p10		FB-F-p5	
		MAE	RMSE	MAE	RMSE
Physics-based model (Sim.)	*L* [dB]	0.25	0.3	0.25	0.32
	*φ* [°]	2.15	2.87	2.26	3.43
Transfer function model (TF model)	*L* [dB]	0.47	0.71	0.44	0.62
	*φ* [°]	1.47	1.87	2.64	3.96

The results indicate that, in certain regions of the frequency characteristics, the conventional transfer-function model achieves comparable or even locally better agreement with the experimental data, while in other regions the proposed physics-based model exhibits superior agreement. Overall, however, the proposed model provides better correspondence with the experimental results over a wider range of frequencies and operating conditions. A key advantage of the proposed physics-based model over the conventional linear model in the form of a second-order transfer function lies in its modular structure and parameterizability. This enables targeted adjustment of individual physical parameters governing the forces acting on the spool, whereas the transfer-function model requires re-identification of its parameters for each new set of boundary conditions.

## Conclusion

In this work, a physics-based model of a proportional valve was developed and validated, providing a detailed understanding of the interactions between electrical, mechanical, and hydraulic phenomena. The model was designed to allow parameterization of individual physical components, and its predictions were systematically compared with experimental measurements.

The main achievements of this work can be summarized as follows:

- Development of the simulation model—A physics-based model of the proportional spool valve was developed, explicitly accounting for all forces acting on the spool. This approach allows independent parameterization of the individual force components.
- Experimental validation—Static and dynamic characteristics were measured on a dedicated hydraulic test rig. These data were used both to validate the model over a wide range of operating conditions.
- Friction parameter identification—A detailed identification of friction parameters was carried out based on experimental measurements performed under both no-flow and flow conditions, in open-loop as well as closed-loop configurations. The results demonstrate that the friction parameters are significantly influenced by the presence of fluid flow and the inlet pressure, and that these effects must be accounted for in order to achieve agreement between simulation and experiment. Furthermore, it was shown that the identified friction parameters are not fully consistent and vary depending on the type of spool motion (step versus harmonic). In particular, the magnitude of the breakaway friction force during step responses was found to depend on the spool velocity, which increases with increasing input voltage.
- Comparison of dynamic characteristics—Numerically obtained step responses and frequency characteristics were compared with experimentally measured data acquired in both feedback and no-feedback configurations, under no-flow conditions as well as with fluid flow at different inlet pressures. The results confirm that the proposed model exhibits good agreement with experimental data across a wide range of operating conditions. The most pronounced discrepancies between simulation and experiment occur at higher step input amplitudes, at higher excitation frequencies, and in the presence of hydrodynamic forces, where the real system exhibits more complex dynamic behavior due to nonlinearities and pressure- and flow-dependent variations in friction phenomena.

The obtained results confirm that the developed model represents a suitable tool for predicting the behavior of proportional valves under various operating conditions and can be applied both to the design and optimization of control algorithms and to the analysis of the influence of individual physical parameters. In addition, the model was compared with a commonly used linear model in the form of a second-order transfer function, which further supports the relevance and practical value of the proposed physics-based modeling approach. Further development of the model should focus particularly on refining the description of friction forces, which have a significant impact on spool motion dynamics. It is advisable to consider the effects of the contact area between the spool and the valve body, as well as the influence of the working fluid temperature on the friction parameters. In the present study, the temperature was kept constant. In real operating conditions, variations in temperature can significantly affect the viscous component of friction.

Future research directions include:

- Refinement of the friction model by incorporating geometric effects (contact area between spool and valve body),
- Modeling the dependence of friction forces on the working fluid temperature,
- A more detailed analysis of friction parameters for different types of spool motion (step and harmonic), with a focus on understanding the physical mechanisms that cause variations in these parameters.

These extensions would improve the accuracy of dynamic behavior predictions and expand the potential applications of the model in the design and optimization of hydraulic system control algorithms.

## Data Availability

The data that support the findings of this study are available from the corresponding author upon reasonable request.
